# Antibiotic discovery throughout the Small World Initiative: A molecular strategy to identify biosynthetic gene clusters involved in antagonistic activity

**DOI:** 10.1002/mbo3.435

**Published:** 2017-01-22

**Authors:** Elizabeth Davis, Tyler Sloan, Krista Aurelius, Angela Barbour, Elijah Bodey, Brigette Clark, Celeste Dennis, Rachel Drown, Megan Fleming, Allison Humbert, Elizabeth Glasgo, Trent Kerns, Kelly Lingro, MacKenzie McMillin, Aaron Meyer, Breanna Pope, April Stalevicz, Brittney Steffen, Austin Steindl, Carolyn Williams, Carmen Wimberley, Robert Zenas, Kristen Butela, Hans Wildschutte

**Affiliations:** ^1^ Department of Biological SciencesBowling Green State UniversityBowling GreenOHUSA; ^2^ Department of BiologySeton Hill UniversityGreensburgPAUSA

**Keywords:** biosynthetic gene cluster, citizen science, pseudomonads, Small World Initiative

## Abstract

The emergence of bacterial pathogens resistant to all known antibiotics is a global health crisis. Adding to this problem is that major pharmaceutical companies have shifted away from antibiotic discovery due to low profitability. As a result, the pipeline of new antibiotics is essentially dry and many bacteria now resist the effects of most commonly used drugs. To address this global health concern, citizen science through the Small World Initiative (SWI) was formed in 2012. As part of SWI, students isolate bacteria from their local environments, characterize the strains, and assay for antibiotic production. During the 2015 fall semester at Bowling Green State University, students isolated 77 soil‐derived bacteria and genetically characterized strains using the 16S rRNA gene, identified strains exhibiting antagonistic activity, and performed an expanded SWI workflow using transposon mutagenesis to identify a biosynthetic gene cluster involved in toxigenic compound production. We identified one mutant with loss of antagonistic activity and through subsequent whole‐genome sequencing and linker‐mediated PCR identified a 24.9 kb biosynthetic gene locus likely involved in inhibitory activity in that mutant. Further assessment against human pathogens demonstrated the inhibition of *Bacillus cereus*,* Listeria monocytogenes*, and methicillin‐resistant *Staphylococcus aureus* in the presence of this compound, thus supporting our molecular strategy as an effective research pipeline for SWI antibiotic discovery and genetic characterization.

## 1 INTRODUCTION

The emergence of bacterial pathogens resistant to all known antibiotics is a global crisis (Frieden, 2013). The overuse of broad‐spectrum antibiotics in clinical and agricultural settings have introduced selective pressures that have influenced the evolution of resistance to most antibiotics (Kuehn, 2014; Price, Koch, & Hungate, 2015; Robinson et al., 2016; Silbergeld, Graham, & Price, 2008). Alarmingly, the first case of colistin resistance, a last resort drug, was recently reported in the U.S. (Mcgann et al., 2016), and the encoding resistance cassette is now mobilized on a plasmid facilitating the ease of transfer to other bacteria (Rolain & Olaitan, 2016; Schwarz & Johnson, 2016; Stoesser, Mathers, Moore, Day, & Crook, 2016). Thus, there is a growing demand for the identification of new effective antibacterial compounds. According to the Centers for Disease Control and Prevention, an estimated 722,000 infections were acquired in U.S. acute care hospitals in 2011; of these**,** 75,000 patients died during their hospitalizations (Magill et al., 2014). Of particular concern from these growing cases are the ESKAPE pathogens (*Enterococcus faecium*,* Staphylococcus aureus*,* Klebsiella pneumoniae*,* Acinetobacter baumannii*,* Pseudomonas aeruginosa*, and *Enterococcus* species), now recognized by the Infectious Disease Society of America as those bacteria posing the most significant risk to public health. The ESKAPE pathogens are responsible for the majority of nosocomial infections in the United States, but treatment options are dwindling in efficacy due to the pathogens’ high levels of antibiotic resistance (Boucher et al., 2009). Moreover, resistance is not confined to nosocomial settings. Environmental strains also encode resistance (Leamer, Clemmons, Jordan, & Pacha, 2013; Rose et al., 2009; Rothrock, Hiett, Guard, & Jackson, 2016) and contribute to life‐threatening community‐acquired infections (Furuya‐Kanamori et al., 2015; Leamer et al., 2013; McKenna, 2008; Schinasi et al., 2014). Escalating the crisis is the shift of major pharmaceutical companies away from antibiotic discovery and synthesis due to low profitability (Pammolli, Magazzini, & Riccaboni, 2011; Scannell, Blanckley, Boldon, & Warrington, 2012). As a result, the pipeline of new antibiotics is dry and bacteria are now resistant to the effects of most commonly used drugs. We are quickly approaching a preantibiotic era during which untreatable bacterial infections will lead to death for many individuals.

To address this worldwide health threat, citizen science through the Small World Initiative (SWI; www.smallworldinitiative.org) was created that implements crowdsourcing of novel antibiotic discovery to undergraduates in an educational setting. First developed by Jo Handelsman at Yale University in 2012, SWI confronts two important problems: first, the growing economic need for more Science, Technology, Engineering, and Math (STEM) graduates (Holdren & Lander, 2012); and second, antibiotic resistance among pathogens which is now a medical issue of utmost importance (Frieden, 2013; Price et al., 2015; Tommasi, Brown, Walkup, Manchester, & Miller, 2015). To help conquer these issues, SWI encourages students to pursue careers in science through teaching microbiology concepts and real hands‐on research. As part of SWI, students isolate bacteria from their local environments, determine antibiotic production among those strains, and utilize chemical techniques to extract compounds for further characterization. Since its formation, SWI has grown to include 150 participating schools across 35 U.S. states and 12 countries. Despite these efforts, SWI workflow involving chemical extraction methods has proven challenging and the biochemical characterization of any isolated antibiotic has yet to be reported. We present our pipeline as an alternative means to advance antibiotic discovery through the identification of biosynthetic gene clusters (BGCs) involved in drug production. To the best of our knowledge, this is the first SWI research manuscript to be published.

During the course of the 2015 fall semester at Bowling Green State University (BGSU) in OH, students in the Introduction to Microbiology class isolated 77 soil‐derived bacterial strains, characterized these isolates using the 16S rRNA gene, and then tested each for antagonistic activity against “safe” relatives (SRs, nondisease causing strains) of the ESKAPE pathogens. Students identified those environmental strains that exhibited antagonistic activity, and performed transposon mutagenesis to identify the putative gene regions involved in toxigenic compound production. A mutant with loss of inhibitory activity was identified. Subsequent whole‐genome sequencing and linker‐mediated PCR by graduate students identified a ~24.9 kb BGC suggesting a role of the encoded products in the antagonistic activity. Further testing against human pathogens showed this product was effective in inhibiting *Bacillus cereus* and methicillin‐resistant *Staphylococcus aureus* (MRSA). Through a proof of concept, we demonstrate, in collaboration with undergraduate and graduate students, this SWI molecular discovery platform is an effective means to identify BGCs involved in the production of antagonistic compounds.

## 2 MATERIAL AND METHODS

### 2.1 Strain isolation and growth conditions

Soil samples were obtained from the campus of Bowling Green State University, OH on August 27th, 2015 from topsoil. One gram of soil was resuspended in 5 ml sterile 0.85% w/v NaCl, homogenized, and serially diluted in sterile 0.85% w/v NaCl and 100 µl was spread plated onto nutrient broth (NB) solid media (BD Difco) with 1.5% w/v agar (BD Difco) and cetrimide solid media (Sigma). Cultures were incubated at 24°C for 48 hr. Single colonies were picked and streaked for isolation. All environmental strains were cultured at 24°C in liquid or on agar NB media. The soil sample from which strain SWI36 was isolated was obtained at GPS coordinates of 41º22′47″ N 83º38′32″ W. Safe relatives of the ESKAPE pathogens including *Bacillus subtilis*,* Escherichia coli* (ATCC 1775), *Acinetobacter baylyi* (ATCC 33305), *Erwinia carotovora*,* Enterobacter aerogenes* (ATCC 51697), and *Pseudomonas putida* were cultured at 24°C for 20 hr prior to the antagonistic assay. Pathogens including *A. baumannii*,* B. cereus*,* Enterobacter cloacae*,* Enterococcus faecalis*,* E. faecium*,* K. pneumoniae*,* Listeria monocytogenes*, and MRSA were grown at 30–37°C for 20 hr and tested for antagonistic activity. For transposon mutagenesis, *Pseudomonas* strain SWI36 was grown in NB and *E. coli* helper strain HB101 was grown in lysate broth (LB) liquid media with 30 µg/ml of chloramphenicol (Cm) and strain CC118 carrying pBAM1 was grown in LB with 50 μg/ml kanamycin (Km) and 150 µg/ml of ampicillin (Amp) as previously described (Martinez‐Garcia, Calles, Arevalo‐Rodriguez, & de Lorenzo, 2011). *E. coli* strains were incubated at 37°C.

### 2.2 Gene sequencing and phylogenetic analysis

For gene sequencing, bacterial strains were streaked onto NB and cultured for 2 days at 24°C and PCR was performed. A colony was used as a genomic DNA template for PCR. Primers targeting the 16S rRNA gene (16S 27 forward primer: 5′‐ AGR GTT TGA TCM TGG CTC A ‐3′; 16S 1492 reverse primer: 5′‐ TAC GGY TAC CTT GTT AYG ACT T ‐3′) were used to amplify and sequence a 1465 bp region. PCR conditions were as follows: 92°C denaturing for 10 s, 50°C annealing for 30 s, elongation at 72°C for 90 s for 29 cycles. A nucleotide alignment was generated from 469 bp of the 16S rRNA gene and a neighbor‐joining tree was then constructed using Jukes‐Cantor nucleotide distance measurement in CLC Main Workbench (CLC bio, Qiagen). Bootstrapping was performed in 100 replicates.

### 2.3 Antagonistic activity

Environmental strains were streaked on NB agar medium and SRs were cultured in NB broth overnight for 20 hr prior to the antagonistic assay. To generate a bacterial lawn, 50 μl of a single SR culture was spread on NB agar plates. Single colonies of environmental strains were patched onto the spread‐plated SR by picking and streaking onto the plate. Strains were cocultured overnight at 24°C. Antagonistic activity was assessed by the presence of a zone of clearing.

### 2.4 Genome sequencing of strain SWI36

Genomic DNA was extracted using the Wizard Genomic DNA Purification Kit (Promega). PacBio sequencing was performed by the University of Delaware DNA Sequencing & Genotyping Center. Genomic DNA was sheared using g‐tube to 20 kb fragments (Covaris). The PacBio libraries were prepared using standard PacBio protocol for 20 kb libraries (20 kb Template Preparation Using BluePippin Size selection system). After BluePippin (Sage Science), size selection from 10 kb average size of the libraries were around 25 kb. Each sample library was sequenced on PacBio RS II instrument with one SMRT cell using P6‐C4 chemistry with 6‐hr movie. The genome was assembled using PacBio HGAP3 (Hierarchical Genome Assembly Process 3). Reads of inserts were filtered by quality 0.8 and read length 1 kb (Chin et al., 2013). All assemblies folded into 1 contig consisting of 6,170,757 bp. The genome is available at JGI IMG OID# 2681813543.

### 2.5 Transposon mutagenesis

Triparental mating was used to deliver the Tn5 mini‐transposon from *E. coli* strain CC118 with helper strain HB101 to *Pseudomonas* strain SWI36 (Martinez‐Garcia et al., 2011). *E. coli* and *Pseudomonas* SWI36 strains were cultured overnight as described above. One ml of each culture was washed and resuspended in 1 ml of 0.85% w/v NaCl and 500 μl of each was mixed in a 1:1:1 ratio. The mating mixture was vortexed and resuspended in 10 μl 0.85% w/v NaCl. The cell suspension was spotted onto a solid NB agar and incubated at 24°C. Following 48 hr incubation at room temperature, the cells were resuspended in 100 μl 0.85% w/v NaCl, diluted 1:10, and plated onto solid cetrimide agar with 50 μg/ml Km to select for *Pseudomonas* transconjugants. Transconjugants were replica‐plated onto 50 μl of the spread plated‐sensitive *Bacillus subtilis* strain, incubated at 24°C for 48 hr, and screened for mutants exhibiting loss of antagonistic phenotype.

### 2.6 Mutant DNA extraction and linker‐mediated PCR

Genomic DNA was extracted from the SWI36 mutant using the Wizard Genomic DNA Purification kit (Promega). Two micrograms of genomic DNA was digested using restriction enzymes *Pvu*II*, Sca*I*, Sma*I*, and Ssp*I from New England Biolabs according to their protocols. The fragmented products were purified using the Nucleospin Gel and PCR Clean‐up kit (Machery‐Nagel). The resulting purified digested DNA was ligated to 4 μmol/µl of annealed linker PCR primers, BPHI (5′ CAA GGA AGG ACG CTG TCT GTC GAA GGT AAG GAA CGG ACG AGA GAA GGG AGA G 3′) and BPHII (5′ CTC TCC CTT TCG AAT CGT AAC CGT TCG TAC GAG AAT CGC TGT CCT CTC CTT G 3′), using T4 DNA ligase. Purification of the ligation was done according to the manufacturer's protocol (Machery‐Nagel). Linker‐mediated (LM) PCR was performed in two cycles. LM‐PCR I was performed using 2 μl of ligated DNA and 5 μmol/L primers 224 (5′ CGA ATC GTA CCG TTC GTA CGA GAA TCG CT 3′) and Tn primer 1, pBAM1 3424 Rev, (5′ ATC CAT GTT GCT GTT CAG AC 3′). PCR conditions for the BPCR I reaction were as follows: 92°C denaturing for 10 sec, 50°C annealing for 60 sec, elongation at 72°C for 90 sec for 19 cycles. One microliter of LM‐PCR I product was used as template for LM‐PCR II using primers 224 (5′ CGA ATC GTA CCG TTC GTA CGA GAA TCG CT 3′) and Tn primer 2 pBAM1 3373 Rev (5′ ATG GCT CAT AAC ACC CCT TG 3′). PCR conditions for the LM‐PCR II reaction were as follows: 92°C for 120 sec, 55°C for 30 sec, and 72°C for 90 sec for 34 cycles. Sequencing was performed using primers 224 and pBAM1 3373 Rev at the University of Chicago Comprehensive Cancer Center DNA Sequencing and Genotyping facility.

## 3 RESULTS AND DISCUSSION

### 3.1 Strategy for strain isolation

**Figure 1:**
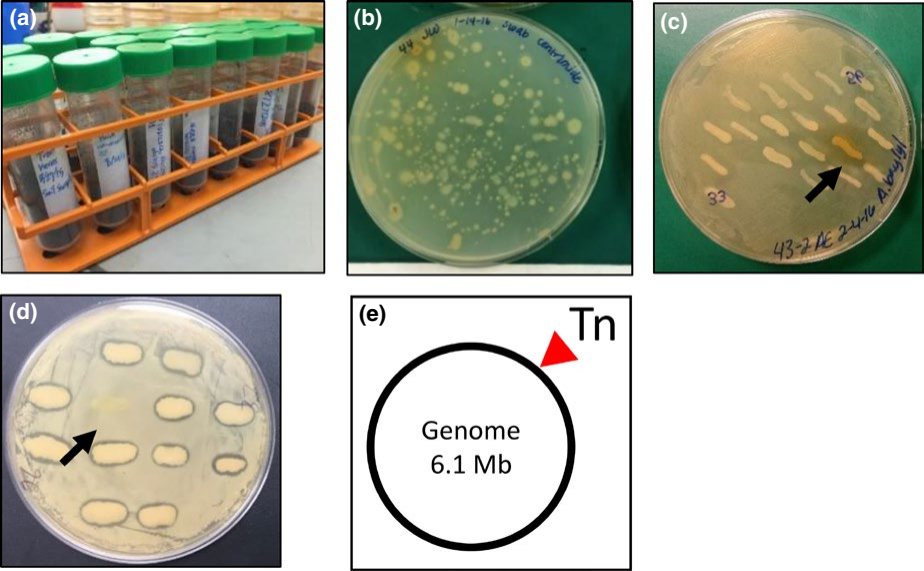
Diagram of the SWI research workflow. (a) Soil samples were collected and (b) bacterial strains were isolated through serial dilution. (c) Competition plate assays were used to determine antagonistic activity among isolates. Strains were co‐grown and observed for antagonistic activity by screening for a zone of clearing (black arrow). (d) Tn mutagenesis was performed to identify mutants (black arrow) showing loss of antagonistic activity. (e) Linker‐mediated PCR and genome sequencing was used to identify the Tn insertion and the BGC likely involved in antagonistic activity.

For our SWI research strategy, pseudomonads were chosen as a model organism to pursue antibiotic discovery because of their high levels of genomic diversity (Gross & Loper, 2009; Silby, Winstanley, Godfrey, Levy, & Jackson, 2011) and persistence in diverse habitats such as soil (Chatterjee et al., 2017; Morris, Monteil, & Berge, 2013), in association with plants (Berendsen, Pieterse, & Bakker, 2012; Bulgarelli et al., 2015; Loper et al., 2012), and within freshwater ecosystems (Chatterjee et al., 2017; D'souza et al., 2013; Morris et al., 2010). Given the unique physical state of these distinct habitats, we reason that strains adapted to different environments should maintain unique metabolic pathways capable of producing diverse secondary metabolites that may exhibit antimicrobial effects against other bacteria. Moreover, pseudomonads have been shown to breakdown refractory recalcitrant compounds such as chloroanilines (Nitisakulkan et al., 2014), insecticides (Pinjari, Pandey, Kamireddy, & Siddavattam, 2013), and chitin (Thompson, Smith, Wilkinson, & Peek, 2001), inhibit the growth of pathogenic plant fungi (Nielsen, Sorensen, Fels, & Pedersen, 1998; Nielsen, Thrane, Christophersen, Anthoni, & Sorensen, 2000; Tran, Ficke, Asiimwe, Hofte, & Raaijmakers, 2007), exhibit antitumor activity (Ikeda et al., 1983; Ni et al., 2009), and inhibit growth of a wide range of bacteria including human pathogens of MRSA (Farrow & Pesci, 2007; Rode, Hanslo, de Wet, Millar, & Cywes, 1989), *Mycobacterium tuberculosis* (Gerard et al., 1997), collections of gram‐positive and gram‐negative bacteria (Ye et al., 2014), and *P. aeruginosa* isolated from cystic fibrosis patients (Chatterjee et al., 2017). Thus, *Pseudomonas* spp. are ideal targets for the SWI antibiotic discovery research platform given their production of diverse natural metabolites and occupancy in unique ecological habitats. Sample locations were selected locally by BGSU undergraduate students (Figure 1a) and immediately processed on NB and cetrimide media. Each student isolated at least one strain (Figure 1b), thus giving a collection of 77 isolates, about half of which were predicted to be pseudomonads based on cetrimide selection.

### 3.2 Phylogenetic characterization and antagonistic activity

**Figure 2:**
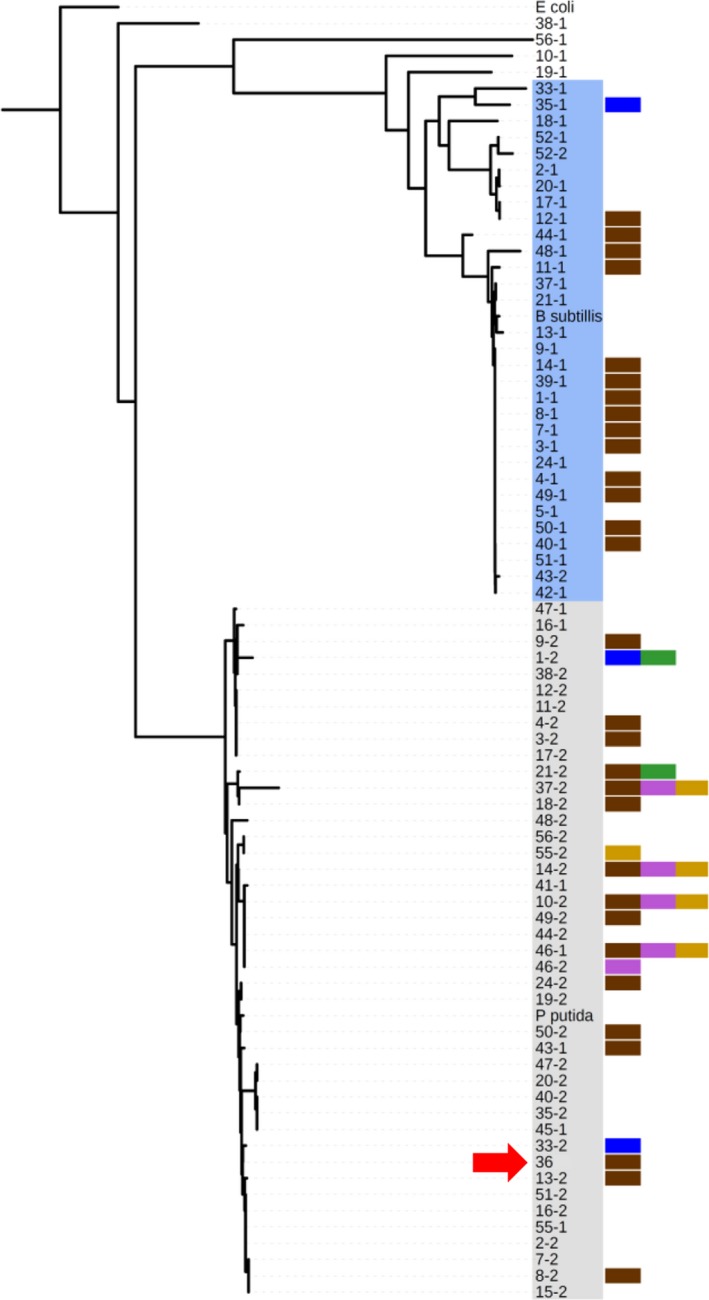
A neighbor‐joining phylogenetic tree based on partial sequence of the 16S rRNA gene of 77 environmental strains was created and overlaid with antagonistic activity results. Colored bars indicate strains in key that were inhibited by environmental isolates (*A. baylyi*, purple; *B. subtilis*, brown; *E. aerogenes*, gold; *E. carotovora*, green; *E. coli K12*, blue; *P. putida* red). *Pseudomonas* strain SWI36 is identified by the red arrow. Gray and blue shaded clades represent *Pseudomonas* and *Bacillus* strains, respectively.

**Figure 3:**
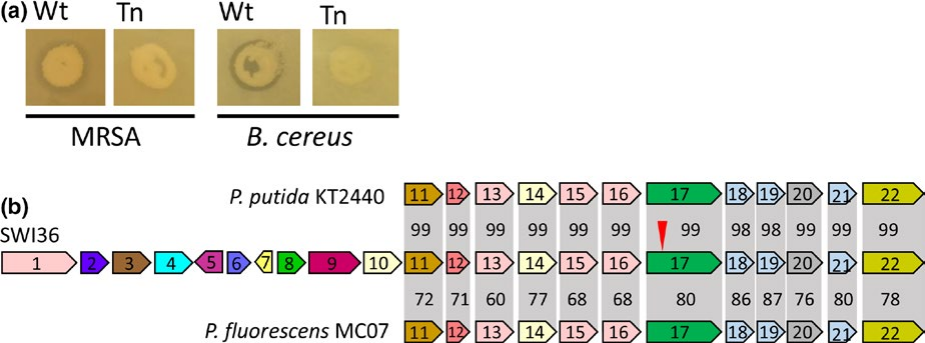
Loss of antagonistic phenotype by transposon insertion in an aminotransferase encoding gene in *Pseudomonas* strain SWI36. (a) Wild‐type and mutant strain showing loss of inhibition phenotype on MRSA and *B. cereus*. (b) The 24.9 kb BGC of SWI36 compared to *P. putida* KT2440 and *P. fluorescens* MC07. The Tn insertion is indicated by the arrow. The 14.7 kb locus content in SWI36 is 99% and 76% similar to the region found in *P. putida* KT2440 and *P. fluorescens* MC07, respectively. Individual ORF amino acid percent similarity is indicated with respect to SWI36 in the shaded gray regions. Gene numbered 1–22 correspond to ORFs listed in Table 1.

For examination of strain diversity, the 16S rRNA gene was amplified, sequenced, and used to construct a neighbor‐joining phylogenetic tree. From our isolation strategy involving selection with cetrimide and growth on NB, two distinct clades comprised of *Pseudomonas* and *Bacillus* strains were identified (Figure 2, shaded clades), in addition to isolates belonging to *Rheinheimera*,* Chryseobacterium*,* Paenibacillus*, and *Lysinibacillus* spp. (Figure 2, unshaded clade). As a means to assess competition, we utilized a plate‐based assay in which strains are co‐grown in one‐to‐one competitions, for which we utilized SRs of ESKAPE pathogens and then screened for antagonistic activity (Figure 1c). We define effective competition by a zone of clearing that extends at least 1 mm from the colony's edge, thus inhibiting the SR. All environmental strains were tested against six SRs including *B. subtilis* (*B. cereus* SR), *Escherichia coli* K12 (*E. coli* O157:H7 SR), *A. baylyi* (*A. baumannii* SR), *Erwinia carotovora* (plant pathogen), *Enterobacter aerogenes* (*E. cloacae* SR), and *Pseudomonas putida* (*P. aeruginosa* SR) which resulted in 462 one‐to‐one bacterial interactions. A total of 33 out of 77 strains (43%) were found to exhibit antagonistic activity toward at least one of the SR isolates (Figure 2). Five strains were able to inhibit multiple SRs including both gram‐positive and gram‐negative isolates suggesting a broad range in antagonistic activity. Strain SWI36 (Figure 2, red arrow) was able to antagonize the SR *B. subtilis* and additional characterization performed by BGSU graduate students showed activity against other human pathogens including *B. cereus*,* L. monocytogenes*, and MRSA suggesting it has broad host activity against gram‐positive pathogens (Figure 3a). Thus, through undergraduate efforts utilizing the SWI platform, we have isolated a collection of environmental strains that are able to directly inhibit several human pathogens.

### 3.3 Identification of a biosynthetic gene cluster involved in antagonistic activity

Traditional SWI workflow utilizes chemical approaches to extract inhibitory compounds from antagonistic strains for their subsequent characterization. Previously, we optimized molecular methods for the identification of BGCs involved in antagonistic activity among pathogenic *P. aeruginosa* strains from cystic fibrosis patients (Chatterjee et al., 2017). We adapted this methodology, involving transposon (Tn) mutagenesis using the pBAM1 vector (Martinez‐Garcia et al., 2011), to SWI in order to expand the process of antibiotic discovery by identifying and characterizing BGCs involved in antagonistic activities. Because SWI36 could inhibit multiple pathogens and was susceptible to conjugation and Tn activity, SWI36 was subjected to a large‐scale mutant hunt during the Fall 2015 SWI course. Using the adapted molecular workflow, undergraduates performed Tn mutagenesis and screened ~10,000 mutants, ultimately identifying one that lost the ability to inhibit the growth of the SR *B. subtilis* (Figure 1d) and human pathogens *B. cereus*,* L. monocytogenes*, and MRSA (Figure 3a). Thus, the scientific rigor of this approach is made evident through the identified antagonistic strains and the generation of a loss of inhibition phenotype mutant for novel BGC discovery (Chatterjee et al., 2017).

To assist in the identification of the Tn insertion, genome sequencing of SWI36 was performed using PacBio technology which yielded a single closed contig of 6.1 Mb. Annotation by the Joint Genome Institute (JGI) revealed that the SWI36 genome encodes 5,492 protein coding genes, of which 4,505 have a predicted function, 167 RNA genes, and 19 BGCs comprising of 286 genes predicted to be involved in secondary metabolite production. Through subsequent linker‐mediated PCR of the Tn flanking region in the SWI36 mutant coupled with genome sequencing of the wild‐type strain, we confirmed the insertion in a gene that encodes a pyridoxal phosphate‐dependent aminotransferase. This gene was localized in a 24.9 kb region identified within the JGI BGC cluster #161750310 and encodes 19 genes (ORFs 1–19 in Figure 3b, Table 1). In addition to the aminotransferase, ORFs 12–19 are predicted to encode an acyl carrier protein, four 3‐oxylacyl synthases, one 3‐oxylacyl reductase, and a hydroxybutyrate dehydrogenase which are characteristic of a type‐II polyketide synthase (Dreier & Khosla, 2000). A LysR transcriptional regulator and transporter are encoded in ORFs 8 and 22, respectively, may control the expression and transport of the compound. The BGC was BLASTed against NCBI and JGI to determine if other bacteria encoded this locus; results showed a 14.7 kb region was 99% similar at the nucleotide level to *Pseudomonas putida* strain KT2440 and 76% similar to *Pseudomonas fluorescens* strain MC07 gene cluster (Figure 3b, ORFs 11–22) that has been shown to encode a product with antifungal activity (Jinwoo et al., 2006). We performed an average nucleotide identity (ANI) comparison between SWI36 and other *Pseudomonas* genomes to determine its relatedness to other strains (Table 2). Similar to the BGC identity, ANI results showed that SWI36 was ~98% and ~97% similar to *P. putida* strains KT2440 and NR1 suggesting SWI36 is a member of the *P. putida* group. Based on loss of activity from Tn mutagenesis and the predicted functions of products involved in a type‐II polyketide synthase, we predict the SWI36 BGC contributes to the production of an antagonistic factor that inhibits the growth of gram‐positive bacteria including the human pathogens *B. cereus*,* L. monocytogenes*, and MRSA.

**Table 1:** Predicted open reading frames identified in SWI36 BGC

ORF	JGI locus tag Ga0131960_	AA length	Predicted protein	Best hit genome
1	114729	832	Dimethylsulfoniopropionate cleavage enzyme	*Burkholderia cepacia* AMMD
2	114730	385	Alcohol dehydrogenase	*Pseudomonas mendocina* ymp
3	114731	498	Malonate‐semialdehyde dehydrogenase	*P. mendocina* ymp
4	114732	545	Betaine/carnitine transporter, BCCT family	*Desulfonispora thiosulfatigenes* GKNTAUT, DSM 11270
5	114733	318	NmrA‐like family protein	*Pseudomonas fluorescens* WH6
6	114734	152	Transcriptional regulator	*P. fluorescens* SBW25
7	114735	79	Transposase	
8	114736	306	LysR transcriptional regulator	*P. putida* N1R
9	114737	484	Tricarballylate dehydrogenase	*P. putida* N1R
10	114738	392	Citrate utilization protein	*P. putida* N1R
11	114739	434	Citrate‐Mg2 + :H+ symporter	*P. putida* S16
12	114740	189	Acyl carrier protein	*P. putida* KT2440
13	114741	424	3‐oxoacyl synthase	*P. putida* KT2440
14	114742	360	3‐oxoacyl synthase	*P. putida* NIR
15	114743	432	3‐oxoacyl synthase II	*P. putida* NIR
16	114744	403	3‐oxoacyl synthase II	*P. putida* KT2440
17	114745	956	Aminotransferase III	*P. putida* KT2440
18	114746	245	3‐oxoacyl reductase	*P. putida* KT2440
19	114747	254	3‐hydroxybutyrate dehydrogenase	*P. putida* KT2440
20	114748	417	Hypothetical protein	*P. putida* KT2440
21	114749	278	Transcriptional regulator	*P. putida* KT2440
22	114750	869	Transporter	*P. putida* KT2440

**Table 2:** Average nucleotide identity between the genomes of *Pseudomonas* strains

	*Pseudomonas* sp. SWI36	*P. putida* KT2440	*P. putida* N1R	*P. putida* S16	*P. putida* W15oct28	*P. protegens* Pf‐5	*P. fluorescens* ATCC 17400	*P. fluorescens* ATCC 13525	*P. aeruginosa* PAO1
*Pseudomonas* sp. SWI36		98.13	97.53	90.00	79.72	79.28	78.52	90.37	77.37
*P. putida* KT2440	98.12		97.33	89.87	90.45	79.32	78.59	78.22	77.37
*P. putida*N1R	97.45	97.30		89.78	90.39	79.26	78.49	78.12	77.28
*P. putida*S16	90.01	89.84	89.80		90.63	79.99	79.09	78.59	78.02
*P. putida* W15oct28	90.38	90.44	90.37	90.66		79.72	78.88	78.47	77.69
*P. protegens*Pf‐5	79.31	79.30	79.26	80.03	79.71		82.59	81.67	78.16
*P. fluorescens* ATCC 17400	78.54	78.59	78.42	79.09	78.88	82.63		85.20	76.95
*P. fluorescens* ATCC 13525	78.23	78.18	78.12	78.58	78.44	81.64	85.21		76.39
*P. aeruginosa* PAO1	77.38	77.38	77.23	77.97	77.68	78.17	76.96	76.36	

At BGSU, we partition SWI into three goals: isolation and identification of soil‐derived bacteria (Figures 1a–b), antagonistic activity and phylogenetic relatedness (Figures 1c–d and 2), and BGC discovery (Figure 1e). Through our modified SWI workflow, we identified a gene region that encodes a putative antibiotic (Figure 3), with the ability to directly and effectively inhibit both wild and pathogenic strains. The SWI platform has proven effective as a teaching strategy, and here, we show its promising scientific rigor for drug discovery through the identification of BGCs involved in antimicrobial production. Through SWI, students perform important research and are challenged with critical thinking problems through experimental design, data gathering, and analysis of results. A recent educational study involving undergraduates at Florida Atlantic University has shown that compared to a control non‐SWI lab class, SWI students achieve higher grades and earned higher critical thinking scores (Caruso, Israel, Rowland, Lovelace, & Saunders, 2016). Thus, SWI students have improved scores compared to traditional lab classes, and they are at the forefront of basic research that has potential for antibiotic discovery and drug development. For research purposes, we expanded the SWI approach to identify BGCs involved in antagonistic activity with the goal to advance drug discovery beyond standard chemical extraction techniques involving a wild‐type isolate alone. Comparisons between wild‐type and SWI36 Tn‐derived mutant extracts using subsequent biochemical techniques can now be utilized to more quickly identify antibiotic compounds. Studies are underway to determine and characterize the novelty of the SWI36 produced compound. The strategy we outline here serves as a proof of concept for SWI antibiotic discovery utilizing a molecular research platform and includes addition STEM interests beyond microbiology, such as topics in molecular biology, genetics, genomics, and bioinformatics that can be directly incorporated into SWI for increased STEM engagement.

## CONFLICT OF INTEREST

None declared.
