# Supplementary material for: Antibiotic discovery throughout the Small World Initiative: A molecular strategy to identify biosynthetic gene clusters involved in antagonistic activity
Source: Microbiologyopen. 2017 Jan 22;6(3):e00435. doi: 10.1002/mbo3.435 (PMC5458470; doi:10.1002/mbo3.435)
Supplement: Supplementary file 1 [file MBO3-6-na-s001.pdf]

16S gene sequences

>1_1
ACCTGCCCATAAGACTGGGATAACTCCGGGAAACCGGGGCTAATACCGGATAATATTTTGAACTGCATGG
TTCGAAATTGAAAGGCGGCTTCGGCTGTCACTTATGGATGGACCCGCGTCGCATTAGCTAGTTGGTGAGG
TAACGGCTCACCAAGGCAACGATGCGTAGCCGACCTGAGAGGGTGATCGGCCACACTGGGACTGAGACAC
GGCCCAGACTCCTACGGGAGGCAGCAGTAGGGAATCTTCCGCAATGGACGAAAGTCTGACGGAGCAACGC
CGCGTGAGTGATGAAGGCTTTCGGGTCGTAAAACTCTGTTGTTAGGGAAGAACAAGTGCTAGTTGAATAA
GCTGGCACCTTGACGGTACCTAACCAGAAAGCCACGGCTAACTACGTGCCAGCAGCCGCGGTAATACGTA
GGTGGCAAGCGTTATCCGGAATTATTGGGCGTAAAGCGCGCGCAGGTGGTTTCTTAAGT

>1_2
ATCTGCCTGGTAGTGGGGGTCTACGTTTCGAAAGGAACGCTAATACCGCATAATATTTCGTCCTACAGGA
TTCGAAAGCAGGGGACCTTCGGGCCTTGCGCTATCAGATGAGCCTAGGTCGGATTAGCTAGTTGGTGAGG
TAATGGCTCACCAAGGCAACGATCCGTAACTGGTCTGAGAGGATGATCAGTCACACTGGAACTGAGACAC
GGTCCAGACTCCTACGGGAGGCAGCAGTGGGGAATATTGGACAATGGGCGAAAGCCTGATCCAGCCATGC
CGCGTGTGTGAAGAAGGTCTTCGGATTGTAAAGCACTTTAAGTTGGGAGGAACGGGTTGTAGATTAATAC
TCTGCAATTTTGACGTTACCGACAGAATAAGCACCGGCTAACTCTGTGCCAGCAGCCGCGGTAATACAGA
GGGGGCAAGCGTTAATCGGAATTACTGGGCGTAAAGCGCGCGTAGGTGGTTCGTTAAGT

>2_1
ACCTGCCTGTAAGACTGGGATAACTTCGGGAAACCGAAGCTAATACCGGATAGGATCTTCTCCTTCATGG
GAGATGATTGAAAGATGGTTTCGGCTATCACTTACNGATGGGCCCGCGGTGCATTAGCTAGTTGGTGAGG
TAACGGCTCACCAAGGCAACGATGCATAGCCGACCTGAGAGGGTGATCGGCCACACTGGGACTGAGACAC
GGCCCAGACTCCTACGGGAGGCAGCAGTAGGGAATCTTCCGCAATGGACGAAAGTCTGACGGAGCAACGC
CGCGTGAGTGATGAAGGCTTTCGGGTCGTAAAACTCTGTTGTTAGGGAAGAACAAGTACGAGAGTAACTA
GCTCGTACCTTGACGGTACCTAACCAGAAAGCCACGGCTAACTACGTGCCAGCAGCCGCGGTAATACGTA
GGTGGCAAGCGTTATCCGGAATTATTGGGCGTAAAGCGCGCGCAGGCGGTTTCTTAAGT

>2_2
ATCTGCCTGGTAGTGGGGGACAACGTTTCGAAAGGAACGCTAATACCGCATACGTCCTACGGGAGAAAGC
AGGGGACCTTCGGGCCTTGCGCTATCAGATGAGCCTAGGTCGGATTAGCTAGTTGGTGGGGTAATGGCTC
ACCAAGGCGACGATCCGTAACTGGTCTGAGAGGATGATCAGTCACACTGGAACTGAGACACGGTCCAGAC
TCCTACGGGAGGCAGCAGTGGGGAATATTGGACAATGGGCGAAAGCCTGATCCAGCCATGCCGCGTGTGT
GAAGAAGGTCTTCGGATTGTAAAGCACTTTAAGTTGGGAGGAAGGGCAGTAAGCGAATACCTTGCTGTTT
TGACGTTACCGACAGAATAAGCACCGGCTAACTCTGTGCCAGCAGCCGCGGTAATACAGAGGGTGCAAGC
GTTAATCGGAATTACTGGGCGTAAAGCGCGCGTAGGTGGTTTGTTAAGT

>3_1
ACCTGCCCATAAGACTGGGATAACTCCGGGAAACCGGGGCTAATACCGGATAATATTTTGAACTGCATGG
TTCGAAATTGAAAGGCGGCTTCGGCTGTCACTTATGGATGGACCCGCGTCGCATTAGCTAGTTGGTGAGG
TAACGGCTCACCAAGGCAACGATGCGTAGCCGACCTGAGAGGGTGATCGGCCACACTGGGACTGAGACAC
GGCCCAGACTCCTACGGGAGGCAGCAGTAGGGAATCTTCCGCAATGGACGAAAGTCTGACGGAGCAACGC
CGCGTGAGTGATGAAGGCTTTCGGGTCGTAAAACTCTGTTGTTAGGGAAGAACAAGTGCTAGTTGAATAA
GCTGGCACCTTGACGGTACCTAACCAGAAAGCCACGGCTAACTACGTGCCAGCAGCCGCGGTAATACGTA
GGTGGCAAGCGTTATCCGGAATTATTGGGCGTAAAGCGCGCGCAGGTGGTTTCTTAAGT

>3_2
ATCTGCCTGGTAGTGGGGGACAACGTTTCGAAAGGAACGCTAATACCGCATACGTCCTACGGGAGAAAGC
AGGGGACCTTCGGGCCTTGCGCTATCAGATGAGCCTAGGTCGGATTAGCTAGTTGGTGAGGTAATGGCTC

ACCAAGGCGACGATCCGTAACTGGTCTGAGAGGATGATCAGTCACACTGGAACTGAGACACGGTCCAGAC
TCCTACGGGAGGCAGCAGTGGGGAATATTGGACAATGGGCGAAAGCCTGATCCAGCCATGCCGCGTGTGT
GAAGAAGGTCTTCGGATTGTAAAGCACTTTAAGTTGGGAGGAAGGGTTGTAGATTAATACTCTGCAATTT
TGACGTTACCGACAGAATAAGCACCGGCTAACTCTGTGCCAGCAGCCGCGGTAATACAGAGGGTGCAAGC
GTTAATCGGAATTACTGGGCGTAAAGCGCGCGTAGGTGGTTTGTTAAGT

>4_1
ACCTGCCCATAAGACTGGGATAACTCCGGGAAACCGGGGCTAATACCGGATAATATTTTGAACTGCATGG
TTCGAAATTGAAAGGCGGCTTCGGCTGTCACTTATGGATGGACCCGCGTCGCATTAGCTAGTTGGTGAGG
TAACGGCTCACCAAGGCAACGATGCGTAGCCGACCTGAGAGGGTGATCGGCCACACTGGGACTGAGACAC
GGCCCAGACTCCTACGGGAGGCAGCAGTAGGGAATCTTCCGCAATGGACGAAAGTCTGACGGAGCAACGC
CGCGTGAGTGATGAAGGCTTTCGGGTCGTAAAACTCTGTTGTTAGGGAAGAACAAGTGCTAGTTGAATAA
GCTGGCACCTTGACGGTACCTAACCAGAAAGCCACGGCTAACTACGTGCCAGCAGCCGCGGTAATACGTA
GGTGGCAAGCGTTATCCGGAATTATTGGGCGTAAAGCGCGCGCAGGTGGTTTCTTAAGT

>4_2
ATCTGCCTGGTAGTGGGGGACAACGTTTCGAAAGGAACGCTAATACCGCATACGTCCTACGGGAGAAAGC
AGGGGACCTTCGGGCCTTGCGCTATCAGATGAGCCTAGGTCGGATTAGCTAGTTGGTGAGGTAATGGCTC
ACCAAGGCGACGATCCGTAACTGGTCTGAGAGGATGATCAGTCACACTGGAACTGAGACACGGTCCAGAC
TCCTACGGGAGGCAGCAGTGGGGAATATTGGACAATGGGCGAAAGCCTGATCCAGCCATGCCGCGTGTGT
GAAGAAGGTCTTCGGATTGTAAAGCACTTTAAGTTGGGAGGAAGGGTTGTAGATTAATACTCTGCAATTT
TGACGTTACCGACAGAATAAGCACCGGCTAACTCTGTGCCAGCAGCCGCGGTAATACAGAGGGTGCAAGC
GTTAATCGGAATTACTGGGCGTAAAGCGCGCGTAGGTGGTTTGTTAAGT

>5_1
ACCTGCCCATAAGACTGGGATAACTCCGGGAAACCGGGGCTAATACCGGATAATATTTTGAACTGCATGG
TTCGAAATTGAAAGGCGGCTTCGGCTGTCACTTATGGATGGACCCGCGTCGCATTAGCTAGTTGGTGAGG
TAACGGCTCACCAAGGCAACGATGCGTAGCCGACCTGAGAGGGTGATCGGCCACACTGGGACTGAGACAC
GGCCCAGACTCCTACGGGAGGCAGCAGTAGGGAATCTTCCGCAATGGACGAAAGTCTGACGGAGCAACGC
CGCGTGAGTGATGAAGGCTTTCGGGTCGTAAAACTCTGTTGTTAGGGAAGAACAAGTGCTAGTTGAATAA
GCTGGCACCTTGACGGTACCTAACCAGAAAGCCACGGCTAACTACGTGCCAGCAGCCGCGGTAATACGTA
GGTGGCAAGCGTTATCCGGAATTATTGGGCGTAAAGCGCGCGCAGGTGGTTTCTTAAGT

>7_1
ACCTGCCCATAAGACTGGGATAACTCCGGGAAACCGGGGCTAATACCGGATAATATTTTGAACTGCATGG
TTCGAAATTGAAAGGCGGCTTCGGCTGTCACTTATGGATGGACCCGCGTCGCATTAGCTAGTTGGTGAGG
TAACGGCTCACCAAGGCAACGATGCGTAGCCGACCTGAGAGGGTGATCGGCCACACTGGGACTGAGACAC
GGCCCAGACTCCTACGGGAGGCAGCAGTAGGGAATCTTCCGCAATGGACGAAAGTCTGACGGAGCAACGC
CGCGTGAGTGATGAAGGCTTTCGGGTCGTAAAACTCTGTTGTTAGGGAAGAACAAGTGCTAGTTGAATAA
GCTGGCACCTTGACGGTACCTAACCAGAAAGCCACGGCTAACTACGTGCCAGCAGCCGCGGTAATACGTA
GGTGGCAAGCGTTATCCGGAATTATTGGGCGTAAAGCGCGCGCAGGTGGTTTCTTAAGT

>7_2
ATCTGCCTGATAGTGGGGGACAACGTTTCGAAAGGAACGCTAATACCGCATACGTCCTACGGGAGAAAGC
AGGGGACCTTCGGGCCTTGCGCTATCAGATGAGCCTAGGTCGGATTAGCTAGTTGGTGGGGTAATGGCTC
ACCAAGGCGACGATCCGTAACTGGTCTGAGAGGATGATCAGTCACACTGGAACTGAGACACGGTCCAGAC
TCCTACGGGAGGCAGCAGTGGGGAATATTGGACAATGGGCGAAAGCCTGATCCAGCCATGCCGCGTGTGT
GAAGAAGGTCTTCGGATTGTAAAGCACTTTAAGTTGGGAGGAAGGGCAGTAAGCGAATACCTTGCTGTTT
TGACGTTACCGACAGAATAAGCACCGGCTAACTCTGTGCCAGCAGCCGCGGTAATACAGAGGGTGCAAGC
GTTAATCGGAATTACTGGGCGTAAAGCGCGCGTAGGTGGTTTGTTAAGT

>8_1

ACCTGCCCATAAGACTGGGATAACTCCGGGAAACCGGGGCTAATACCGGATAATATTTTGAACTGCATGG
TTCGAAATTGAAAGGCGGCTTCGGCTGTCACTTATGGATGGACCCGCGTCGCATTAGCTAGTTGGTGAGG
TAACGGCTCACCAAGGCAACGATGCGTAGCCGACCTGAGAGGGTGATCGGCCACACTGGGACTGAGACAC
GGCCCAGACTCCTACGGGAGGCAGCAGTAGGGAATCTTCCGCAATGGACGAAAGTCTGACGGAGCAACGC
CGCGTGAGTGATGAAGGCTTTCGGGTCGTAAAACTCTGTTGTTAGGGAAGAACAAGTGCTAGTTGAATAA
GCTGGCACCTTGACGGTACCTAACCAGAAAGCCACGGCTAACTACGTGCCAGCAGCCGCGGTAATACGTA
GGTGGCAAGCGTTATCCGGAATTATTGGGCGTAAAGCGCGCGCAGGTGGTTTCTTAAGT

>8_2
ATCTGCCTGATAGTGGGGGACAACGTTTCGAAAGGAACGCTAATACCGCATACGTCCTACGGGAGAAAGC
AGGGGACCTTCGGGCCTTGCGCTATCAGATGAGCCTAGGTCGGATTAGCTAGTTGGTGGGGTAATGGCTC
ACCAAGGCGACGATCCGTAACTGGTCTGAGAGGATGATCAGTCACACTGGAACTGAGACACGGTCCAGAC
TCCTACGGGAGGCAGCAGTGGGGAATATTGGACAATGGGCGAAAGCCTGATCCAGCCATGCCGCGTGTGT
GAAGAAGGTCTTCGGATTGTAAAGCACTTTAAGTTGGGAGGAAGGGCAGTAAGCGAATACCTTGCTGTTT
TGACGTTACCGACAGAATAAGCACCGGCTAACTCTGTGCCAGCAGCCGCGGTAATACAGAGGGTGCAAGC
GTTAATCGGAATTACTGGGCGTAAAGCGCGCGTAGGTGGTTTGTTAAGT

>9_1
ACCTGCCCATAAGACTGGGATAACTCCGGGAAACCGGGGCTAATACCGGATAATATTTTGAACTGCATGG
TTCGAAATTGAAAGGCGGCTTCGGCTGTCACTTATGGATGGACCCGCGTCGCATTAGCTAGTTGGTGAGG
TAACGGCTCACCAAGGCAACGATGCGTAGCCGACCTGAGAGGGTGATCGGCCACACTGGGACTGAGACAC
GGCCCAGACTCCTACGGGAGGCAGCAGTAGGGAATCTTCCGCAATGGACGAAAGTCTGACGGAGCAACGC
CGCGTGAGTGATGAAGGCTTTCGGGTCGTAAAACTCTGTTGTTAGGGAAGAACAAGTGCTAGTTGAATAA
GCTGGCACCTTGACGGTACCTAACCAGAAAGCCACGGCTAACTACGTGCCAGCAGCCGCGGTAATACGTA
GGTGGCAAGCGTTATCCGGAATTATTGGGCGTAAAGCGCGCGCAGGTGGTTTCTTAAGT

>9_2
ATCTGCCTGGTAGTGGGGGACAACGTTTCGAAAGGAACGCTAATACCGCATACGTCCTACGGGAGAAAGC
AGGGGACCTTCGGGCCTTGCGCTATCAGATGAGCCTAGGTCGGATTAGCTAGTTGGTGAGGTAATGGCTC
ACCAAGGCGACGATCCGTAACTGGTCTGAGAGGATGATCAGTCACACTGGAACTGAGACACGGTCCAGAC
TCCTACGGGAGGCAGCAGTGGGGAATATTGGACAATGGGCGAAAGCCTGATCCAGCCATGCCGCGTGTGT
GAAGAAGGTCTTCGGATTGTAAAGCACTTTAAGTTGGGAGGAAGGGTTGTAGATTAATACTCTGCAATTT
TGACGTTACCGACAGAATAAGCACCGGCTAACTCTGTGCCAGCAGCCGCGGTAATACAGAGGGTGCAAGC
GTTAATCGGAATTACTGGGCGTAAAGCGCGCGTAGGTGGTTCGTTAAGT

>10_1
ACCTGCCCTTCAGACTGGGATAACTACCGGAAACGGTAGCTAATACCGGATAATTTCTTTTTCTCCTGA
GAGAAGAATGAAAGACGGAGCAATCTGTCACTGAGGGATGGGCCTGCGGCGCATTAGCTAGTTGGTGGGG
TAACGGCCCACCAAGGCGACGATGCGTAGCCGACCTGAGAGGGTGAACGGCCACACTGGGACTGAGACAC
GGCCCAGACTCCTACGGGAGGCAGCAGTAGGGAATCTTCCGCAATGGGCGAAAGCCTGACGGAGCAACGC
CGCGTGAGTGATGAAGGTTTTCGGATCGTAAAGCTCTGTTGCCAGGGAAGAACGTCCGGTAGAGTAACTA
GCTACCGGAGTGACGGTACCTGAGAAGAAAGCCCCGGCTAACTACGTGCCAGCAGCCGCGGTAATACGTA
GGGGGCAAGCGTTGTCCGGAATTATTGGGCGTAAAGCGCGCGCAGGCGGCTATTTAAGT

>10_2
ATCTGCCTGGTAGTGGGGGACAACGTTTCGAAAGGAACGCTAATACCGCATACGTCCTACGGGAGAAAGC
AGGGGACCTTCGGGCCTTGCGCTATCAGATGAGCCTAGGTCGGATTAGCTAGTAGGTGAGGTAATGGCTC
ACCTAGGCGACGATCCGTAACTGGTCTGAGAGGATGATCAGTCACACTGGAACTGAGACACGGTCCAGAC
TCCTACGGGAGGCAGCAGTGGGGAATATTGGACAATGGGCGAAAGCCTGATCCAGCCATGCCGCGTGTGT
GAAGAAGGTCTTCGGATTGTAAAGCACTTTAAGTTGGGAGGAAGGGCAGTAAGTTAATACCTTGCTGTTT
TGACGTTACCGACAGAATAAGCACCGGCTAACTCTGTGCCAGCAGCCGCGGTAATACAGAGGGTGCAAGC
GTTAATCGGAATTACTGGGCGTAAAGCGCGCGTAGGTGGTTCGTTAAGT

>11_1
ACCTACCCATAAGACTGGGATAACTCCGGGAAACCGGGGCTAATACCGGATAATATTTTGAACTGCATAG
TTCGAAATTGAAAGGCGGCTTCGGCTGTCACTTATGGATGGACCCGCGTCGCATTAGCTAGTTGGTGAGG
TAACGGCTCACCAAGGCGACGATGCGTAGCCGACCTGAGAGGGTGATCGGCCACACTGGGACTGAGACAC
GGCCCAGACTCCTACGGGAGGCAGCAGTAGGGAATCTTCCGCAATGGACGAAAGTCTGACGGAGCAACGC
CGCGTGAGTGATGAAGGCTTTCGGGTCGTAAAACTCTGTTGTTAGGGAAGAACAAGTGCTAGTTGAATAA
GCTGGCACCTTGACGGTACCTAACCAGAAAGCCACGGCTAACTACGTGCCAGCAGCCGCGGTAATACGTA
GGTGGCAAGCGTTATCCGGAATTATTGGGCGTAAAGCGCGCGCAGGTGGTTTCTTAAGT

>11_2
ATCTGCCTGGTAGTGGGGGACAACGTTTCGAAAGGAACGCTAATACCGCATACGTCCTACGGGAGAAAGC
AGGGGACCTTCGGGCCTTGCGCTATCAGATGAGCCTAGGTCGGATTAGCTAGTTGGTGAGGTAATGGCTC
ACCAAGGCGACGATCCGTAACTGGTCTGAGAGGATGATCAGTCACACTGGAACTGAGACACGGTCCAGAC
TCCTACGGGAGGCAGCAGTGGGGAATATTGGACAATGGGCGAAAGCCTGATCCAGCCATGCCGCGTGTGT
GAAGAAGGTCTTCGGATTGTAAAGCACTTTAAGTTGGGAGGAAGGGTTGTAGATTAATACTCTGCAATTT
TGACGTTACCGACAGAATAAGCACCGGCTAACTCTGTGCCAGCAGCCGCGGTAATACAGAGGGTGCAAGC
GTTAATCGGAATTACTGGGCGTAAAGCGCGCGTAGGTGGTTTGTTAAGT

>12_1
ACCTGCCTGTAAGACTGGGATAACTTCGGGAAACCGAAGCTAATACCGGATAGGATCTTCTCCTTCATGG
GAGATGATTGAAAGATGGTTTCGGCTATCACTTACAGATGGGCCCGCGGTGCATTAGCTAGTTGGTGAGG
TAACGGCTCACCAAGGCAACGATGCATAGCCGACCTGAGAGGGTGATCGGCCACACTGGGACTGAGACAC
GGCCCAGACTCCTACGGGAGGCAGCAGTAGGGAATCTTCCGCAATGGACGAAAGTCTGACGGAGCAACGC
CGCGTGAGTGATGAAGGCTTTCGGGTCGTAAAACTCTGTTGTTAGGGAAGAACAAGTACAAGAGTAACTA
GCTTGTACCTTGACGGTACCTAACCAGAAAGCCACGGCTAACTACGTGCCAGCAGCCGCGGTAATACGTA
GGTGGCAAGCGTTATCCGGAATTATTGGGCGTAAAGCGCGCGCAGGCGGTTTCTTAAGT

>12_2
ATCTGCCTGGTAGTGGGGGACAACGTTTCGAAAGGAACGCTAATACCGCATACGTCCTACGGGAGAAAGC
AGGGGACCTTCGGGCCTTGCGCTATCAGATGAGCCTAGGTCGGATTAGCTAGTTGGTGAGGTAATGGCTC
ACCAAGGCGACGATCCGTAACTGGTCTGAGAGGATGATCAGTCACACTGGAACTGAGACACGGTCCAGAC
TCCTACGGGAGGCAGCAGTGGGGAATATTGGACAATGGGCGAAAGCCTGATCCAGCCATGCCGCGTGTGT
GAAGAAGGTCTTCGGATTGTAAAGCACTTTAAGTTGGGAGGAAGGGTTGTAGATTAATACTCTGCAATTT
TGACGTTACCGACAGAATAAGCACCGGCTAACTCTGTGCCAGCAGCCGCGGTAATACAGAGGGTGCAAGC
GTTAATCGGAATTACTGGGCGTAAAGCGCGCGTAGGTGGTTTGTTAAGT

>13_1
ACCTGCCCATAAGACTGGGATAACTCCGGGAAACCGGGGCTAATACCGGATAACATTTTGCACCGCATGG
TGCGAAATTCAAAGGCGGCTTCGGCTGTCACTTATGGATGGACCCGCGTCGCATTAGCTAGTTGGTGAGG
TAACGGCTCACCAAGGCAACGATGCGTAGCCGACCTGAGAGGGTGATCGGCCACACTGGGACTGAGACAC
GGCCCAGACTCCTACGGGAGGCAGCAGTAGGGAATCTTCCGCAATGGACGAAAGTCTGACGGAGCAACGC
CGCGTGAGTGATGAAGGCTTTCGGGTCGTAAAACTCTGTTGTTAGGGAAGAACAAGTGCTAGTTGAATAA
GCTGGCACCTTGACGGTACCTAACCAGAAAGCCACGGCTAACTACGTGCCAGCAGCCGCGGTAATACGTA
GGTGGCAAGCGTTATCCGGAATTATTGGGCGTAAAGCGCGCGCAGGTGGTTTCTTAAGT

>13_2
ATCTGCCTGGTAGTGGGGGACAACGTTTCGAAAGGAACGCTAATACCGCATACGTCCTACGGGAGAAAGC
AGGGGACCTTCGGGCCTTGCGCTATCAGATGAGCCTAGGTCGGATTAGCTAGTTGGTGAGGTAATGGCTC
ACCAAGGCGACGATCCGTAACTGGTCTGAGAGGATGATCAGTCACACTGGAACTGAGACACGGTCCAGAC
TCCTACGGGAGGCAGCAGTGGGGAATATTGGACAATGGGCGAAAGCCTGATCCAGCCATGCCGCGTGTGT
GAAGAAGGTCTTCGGATTGTAAAGCACTTTAAGTTGGGAGGAAGGGCAGTAAGCGAATACCTTGCTGTTT

TGACGTTACCGACAGAATAAGCACCGGCTAACTCTGTGCCAGCAGCCGCGGTAATACAGAGGGTGCAAGC
GTTAATCGGAATTACTGGGCGTAAAGCGCGCGTAGGTGGTTTGTTAAGT

>14_1
ACCTGCCCATAAGACTGGGATAACTCCGGGAAACCGGGGCTAATACCGGATAATATTTTGAACTGCATGG
TTCGAAATTGAAAGGCGGCTTCGGCTGTCACTTATGGATGGACCCGCGTCGCATTAGCTAGTTGGTGAGG
TAACGGCTCACCAAGGCAACGATGCGTAGCCGACCTGAGAGGGTGATCGGCCACACTGGGACTGAGACAC
GGCCCAGACTCCTACGGGAGGCAGCAGTAGGGAATCTTCCGCAATGGACGAAAGTCTGACGGAGCAACGC
CGCGTGAGTGATGAAGGCTTTCGGGTCGTAAAACTCTGTTGTTAGGGAAGAACAAGTGCTAGTTGAATAA
GCTGGCACCTTGACGGTACCTAACCAGAAAGCCACGGCTAACTACGTGCCAGCAGCCGCGGTAATACGTA
GGTGGCAAGCGTTATCCGGAATTATTGGGCGTAAAGCGCGCGCAGGTGGTTTCTTAAGT

>14_2
ATCTGCCTGGTAGTGGGGGACAACGTTTCGAAAGGAACGCTAATACCGCATACGTCCTACGGGAGAAAGC
AGGGGACCTTCGGGCCTTGCGCTATCAGATGAGCCTAGGTCGGATTAGCTAGTTGGTGAGGTAATGGCTC
ACCAAGGCGACGATCCGTAACTGGTCTGAGAGGATGATCAGTCACACTGGAACTGAGACACGGTCCAGAC
TCCTACGGGAGGCAGCAGTGGGGAATATTGGACAATGGGCGAAAGCCTGATCCAGCCATGCCGCGTGTGT
GAAGAAGGTCTTCGGATTGTAAAGCACTTTAAGTTGGGAGGAAGGGCAGTAAGTTAATACCTTGCTGTTT
TGACGTTACCGACAGAATAAGCACCGGCTAACTCTGTGCCAGCAGCCGCGGTAATACAGAGGGTGCAAGC
GTTAATCGGAATTACTGGGCGTAAAGCGCGCGTAGGTGGTTCGTTAAGT

>15_2
ATCTGCCTGATAGTGGGGGACAACGTTTCGAAAGGAACGCTAATACCGCATACGTCCTACGGGAGAAAGC
AGGGGACCTTCGGGCCTTGCGCTATCAGATGAGCCTAGGTCGGATTAGCTAGTTGGTGGGGTAATGGCTC
ACCAAGGCGACGATCCGTAACTGGTCTGAGAGGATGATCAGTCACACTGGAACTGAGACACGGTCCAGAC
TCCTACGGGAGGCAGCAGTGGGGAATATTGGACAATGGGCGAAAGCCTGATCCAGCCATGCCGCGTGTGT
GAAGAAGGTCTTCGGATTGTAAAGCACTTTAAGTTGGGAGGAAGGGCAGTAAGCGAATACCTTGCTGTTT
TGACGTTACCGACAGAATAAGCACCGGCTAACTCTGTGCCAGCAGCCGCGGTAATACAGAGGGTGCAAGC
GTTAATCGGAATTACTGGGCGTAAAGCGCGCGTAGGTGGTTTGTTAAGT

>16_1
ATCTGCCTGGTAGTGGGGGACAACGTCTCGAAAGGGACGCTAATACCGCATACGTCCTACGGGAGAAAGC
AGGGGACCTTCGGGCCTTGCGCTATCAGATGAGCCTAGGTCGGATTAGCTAGTTGGTGAGGTAATGGCTC
ACCAAGGCGACGATCCGTAACTGGTCTGAGAGGATGATCAGTCACACTGGAACTGAGACACGGTCCAGAC
TCCTACGGGAGGCAGCAGTGGGGAATATTGGACAATGGGCGAAAGCCTGATCCAGCCATGCCGCGTGTGT
GAAGAAGGTCTTCGGATTGTAAAGCACTTTAAGTTGGGAGGAAGGGTTGTAGATTAATACTCTGCAATTT
TGACGTTACCGACAGAATAAGCACCGGCTAACTCTGTGCCAGCAGCCGCGGTAATACAGAGGGTGCAAGC
GTTAATCGGAATTACTGGGCGTAAAGCGCGCGTAGGTGGTTCGTTAAGT

>16_2
ATCTGCCTGGTAGTGGGGGACAACGTTTCGAAAGGAACGCTAATACCGCATACGTCCTACGGGAGAAAGC
AGGGGACCTTCGGGCCTTGCGCTATCAGATGAGCCTAGGTCGGATTAGCTAGTTGGTGGGGTAATGGCTC
ACCAAGGCGACGATCCGTAACTGGTCTGAGAGGATGATCAGTCACACTGGAACTGAGACACGGTCCAGAC
TCCTACGGGAGGCAGCAGTGGGGAATATTGGACAATGGGCGAAAGCCTGATCCAGCCATGCCGCGTGTGT
GAAGAAGGTCTTCGGATTGTAAAGCACTTTAAGTTGGGAGGAAGGGCAGTAAGCGAATACCTTGCTGTTT
TGACGTTACCGACAGAATAAGCACCGGCTAACTCTGTGCCAGCAGCCGCGGTAATACAGAGGGTGCAAGC
GTTAATCGGAATTACTGGGCGTAAAGCGCGCGTAGGTGGTTTGTTAAGT

>17_1
ACCTGCCTGTAAGACTGGGATAACTTCGGGAAACCGAAGCTAATACCGGATAGGATCTTCTCCTTCATGG
GAGATGATTGAAAGATGGTTTCGGCTATCACTTACAGATGGGCCCGCGGTGCATTAGCTAGTTGGTGAGG
TAACGGCTCACCAAGGCAACGATGCATAGCCGACCTGAGAGGGTGATCGGCCACACTGGGACTGAGACAC

GGCCCAGACTCCTACGGGAGGCAGCAGTAGGGAATCTTCCGCAATGGACGAAAGTCTGACGGAGCAACGC
CGCGTGAGTGATGAAGGCTTTCGGGTCGTAAAACTCTGTTGTTAGGGAAGAACAAGTACAAGAGTAACTA
GCTTGTACCTTGACGGTACCTAACCAGAAAGCCACGGCTAACTACGTGCCAGCAGCCGCGGTAATACGTA
GGTGGCAAGCGTTATCCGGAATTATTGGGCGTAAAGCGCGCGCAGGCGGTTTCTTAAGT

>17_2
ATCTGCCTGGTAGTGGGGGACAACGTTTCGAAAGGAACGCTAATACCGCATACGTCCTACGGGAGAAAGC
AGGGGACCTTCGGGCCTTGCGCTATCAGATGAGCCTAGGTCGGATTAGCTAGTTGGTGAGGTAATGGCTC
ACCAAGGCGACGATCCGTAACTGGTCTGAGAGGATGATCAGTCACACTGGAACTGAGACACGGTCCAGAC
TCCTACGGGAGGCAGCAGTGGGGAATATTGGACAATGGGCGAAAGCCTGATCCAGCCATGCCGCGTGTGT
GAAGAAGGTCTTCGGATTGTAAAGCACTTTAAGTTGGGAGGAAGGGTTGTAGATTAATACTCTGCAATTT
TGACGTTACCGACAGAATAAGCACCGGCTAACTCTGTGCCAGCAGCCGCGGTAATACAGAGGGTGCAAGC
GTTAATCGGAATTACTGGGCGTAAAGCGCGCGTAGGTGGTTTGTTAAGT

>18_1
ACCTGCCTATAAGACTGGGATAACTTCGGGAAACCGGAGCTAATACCGGATACGTTCTTTTCTCGCATGA
GAGAAGATGGAAAGACGGTTTACGCTGTCACTTATAGATGGGCCCGCGGCGCATTAGCTAGTTGGTGAGG
TAATGGCTCACCAAGGCGACGATGCGTAGCCGACCTGAGAGGGTGATCGGCCACACTGGGACTGAGACAC
GGCCCAGACTCCTACGGGAGGCAGCAGTAGGGAATCTTCCGCAATGGACGAAAGTCTGACGGAGCAACGC
CGCGTGAACGAAGAAGGCCTTCGGGTCGTAAAGTTCTGTTGTTAGGGAAGAACAAGTACCAGAGTAACTA
GCTGGTACCTTGACGGTACCTAACCAGAAAGCCACGGCTAACTACGTGCCAGCAGCCGCGGTAATACGTA
GGTGGCAAGCGTTGTCCGGAATTATTGGGCGTAAAGCGCGCGCAGGTGGTTCCTTAAGT

>18_2
ATCTGCCTGGTAGTGGGGGACAACGTTTCGAAAGGAACGCTAATACCGCATACGTCCTACGGGAGAAAGC
AGGGGACCTTCGGGCCTTGCGCTATCAGATGAGCCTAGGTCGGATTAGCTAGTAGGTGAGGTAATGGCTC
ACCTAGGCGACGATCCGTAACTGGTCTGAGAGGATGATCAGTCACACTGGAACTGAGACACGGTCCAGAC
TCCTACGGGAGGCAGCAGTGGGGAATATTGGACAATGGGCGAAAGCCTGATCCAGCCATGCCGCGTGTGT
GAAGAAGGTCTTCGGATTGTAAAGCACTTTAAGTTGGGAGGAAGGGTTGTACGTTAATACCGTGCAATTT
TGACGTTACCGACAGAATAAGCACCGGCTAACTCTGTGCCAGCAGCCGCGGTAATACAGAGGGTGCAAGC
GTTAATCGGAATTACTGGGCGTAAAGCGCGCGTAGGTGGTTTGTTAAGT

>19_1
ACCTACCTTATAGTTTGGGATAACTCCGGGAAACCGGGGCTAATACCGAATAATCTATTTCCTCTCATGA
GGAAATACTGAAAGACGGTTTCGGCTGTCACTATAAGATGGGCCCGCGGCGCATTAGCTAGTTGGTGAGG
TAACGGCTCACCAAGGCGACGATGCGTAGCCGACCTGAGAGGGTGATCGGCCACACTGGGACTGAGACAC
GGCCCAGACTCCTACGGGAGGCAGCAGTAGGGAATCTTCCACAATGGACGAAAGTCTGATGGAGCAACGC
CGCGTGAGTGAAGAAGGATTTCGGTTCGTAAAACTCTGTTGTAAGGGAAGAACAAGTACAGTAGTAACTG
GCTGGTACCTTGACGGTACCTTATTAGAAAGCCACGGCTAACTACGTGCCAGCAGCCGCGGTAATACGTA
GGTGGCAAGCGTTGTCCGGAATTATTGGGCGTAAAGCGCGCGCAGGTGGTTTCTTAAGT

>19_2
ATCTGCCTGGTAGTGGGGGACAACGTTTCGAAAGGAACGCTAATACCGCATACGTCCTACGGGAGAAAGC
AGGGGACCTTCGGGCCTTGCGCTATCAGATGAGCCTAGGTCGGATTAGCTAGTTGGTGGGGTAATGGCTC
ACCAAGGCGACGATCCGTAACTGGTCTGAGAGGATGATCAGTCACACTGGAACTGAGACACGGTCCAGAC
TCCTACGGGAGGCAGCAGTGGGGAATATTGGACAATGGGCGAAAGCCTGATCCAGCCATGCCGCGTGTGT
GAAGAAGGTCTTCGGATTGTAAAGCACTTTAAGTTGGGAGGAAGGGCAGTAAGTTAATACCTTGCTGTTT
TGACGTTACCGACAGAATAAGCACCGGCTAACTCTGTGCCAGCAGCCGCGGTAATACAGAGGGTGCAAGC
GTTAATCGGAATTACTGGGCGTAAAGCGCGCGTAGGTGGTTCGTTAAGT

>20_1

ACCTGCCTGTAAGACTGGGATAACTTCGGGAAACCGAAGCTAATACCGGATAGGATCTTCTCCTTCATGG
GAGATGATTGAAAGATGGTTTCGGCTATCACTTACAGATGGGCCCGCGGTGCATTAGCTAGTTGGTGAGG
TAACGGCTCACCAAGGCAACGATGCATAGCCGACCTGAGAGGGTGATCGGCCACACTGGGACTGAGACAC
GGCCCAGACTCCTACGGGAGGCAGCAGTAGGGAATCTTCCGCAATGGACGAAAGTCTGACGGAGCAACGC
CGCGTGAGTGATGAAGGCTTTCGGGTCGTAAAACTCTGTTGTTAGGGAAGAACAAGTACGAGAGTAACTA
GCTCGTACCTTGACGGTACCTAACCAGAAAGCCACGGCTAACTACGTGCCAGCAGCCGCGGTAATACGTA
GGTGGCAAGCGTTATCCGGAATTATTGGGCGTAAAGCGCGCGCAGGCGGTTTCTTAAGT

>20_2
ATCTGCCTGGTAGTGGGGGACAACGTTTCGAAAGGAACGCTAATACCGCATACGTCCTACGGGAGAAAGC
AGGGGACCTTCGGGCCTTGCGCTATCAGATGAGCCTAGGTCGGATTAGCTAGTTGGTGAGGTAATGGCTC
ACCAAGGCGACGATCCGTAACTGGTCTGAGAGGATGATCAGTCACACTGGAACTGAGACACGGTCCAGAC
TCCTACGGGAGGCAGCAGTGGGGAATATTGGACAATGGGCGAAAGCCTGATCCAGCCATGCCGCGTGTGT
GAAGAAGGTCTTCGGATTGTAAAGCACTTTAAGTTGGGAGGAAGGGCATTAACCTAATACGTTAGTGTTT
TGACGTTACCGACAGAATAAGCACCGGCTAACTCTGTGCCAGCAGCCGCGGTAATACAGAGGGTGCAAGC
GTTAATCGGAATTACTGGGCGTAAAGCGCGCGTAGGTGGTTTGTTAAGT

>21_1
ACCTGCCCATAAGACTGGGATAACTCCGGGAAACCGGGGCTAATACCGGATAACATTTTGAACTGCATGG
TTCGAAATTGAAAGGCGGCTTCGGCTGTCACTTATGGATGGACCCGCGTCGCATTAGCTAGTTGGTGAGG
TAACGGCTCACCAAGGCAACGATGCGTAGCCGACCTGAGAGGGTGATCGGCCACACTGGGACTGAGACAC
GGCCCAGACTCCTACGGGAGGCAGCAGTAGGGAATCTTCCGCAATGGACGAAAGTCTGACGGAGCAACGC
CGCGTGAGTGATGAAGGCTTTCGGGTCGTAAAACTCTGTTGTTAGGGAAGAACAAGTGCTAGTTGAATAA
GCTGGCACCTTGACGGTACCTAACCAGAAAGCCACGGCTAACTACGTGCCAGCAGCCGCGGTAATACGTA
GGTGGCAAGCGTTATCCGGAATTATTGGGCGTAAAGCGCGCGCAGGTGGTTTCTTAAGT

>21_2
ATCTGCCTGGTAGTGGGGGACAACGTTTCGAAAGGAACGCTAATACCGCATACGTCCTACGGGAGAAAGC
AGGGGACCTTCGGGCCTTGCGCTATCAGATGAGCCTAGGTCGGATTAGCTAGTAGGTGAGGTAATGGCTC
ACCTAGGCGACGATCCGTAACTGGTCTGAGAGGATGATCAGTCACACTGGAACTGAGACACGGTCCAGAC
TCCTACGGGAGGCAGCAGTGGGGAATATTGGACAATGGGCGAAAGCCTGATCCAGCCATGCCGCGTGTGT
GAAGAAGGTCTTCGGATTGTAAAGCACTTTAAGTTGGGAGGAAGGGTTGTACGCTAATACCGTGCAATTT
TGACGTTACCGACAGAATAAGCACCGGCTAACTCTGTGCCAGCAGCCGCGGTAATACAGAGGGTGCAAGC
GTTAATCGGAATTACTGGGCGTAAAGCGCGCGTAGGTGGTTTGTTAAGT

>24_1
ACCTGCCCATAAGACTGGGATAACTCCGGGAAACCGGGGCTAATACCGGATAATATTTTGAACTGCATGG
TTCGAAATTGAAAGGCGGCTTCGGCTGTCACTTATGGATGGACCCGCGTCGCATTAGCTAGTTGGTGAGG
TAACGGCTCACCAAGGCAACGATGCGTAGCCGACCTGAGAGGGTGATCGGCCACACTGGGACTGAGACAC
GGCCCAGACTCCTACGGGAGGCAGCAGTAGGGAATCTTCCGCAATGGACGAAAGTCTGACGGAGCAACGC
CGCGTGAGTGATGAAGGCTTTCGGGTCGTAAAACTCTGTTGTTAGGGAAGAACAAGTGCTAGTTGAATAA
GCTGGCACCTTGACGGTACCTAACCAGAAAGCCACGGCTAACTACGTGCCAGCAGCCGCGGTAATACGTA
GGTGGCAAGCGTTATCCGGAATTATTGGGCGTAAAGCGCGCGCAGGTGGTTTCTTAAGT

>24_2
ATCTGCCTGGTAGTGGGGGACAACGTTTCGAAAGGAACGCTAATACCGCATACGTCCTACGGGAGAAAGC
AGGGGACCTTCGGGCCTTGCGCTATCAGATGAGCCTAGGTCGGATTAGCTAGTTGGTGGGGTAATGGCTC
ACCAAGGCGACGATCCGTAACTGGTCTGAGAGGATGATCAGTCACACTGGAACTGAGACACGGTCCAGAC
TCCTACGGGAGGCAGCAGTGGGGAATATTGGACAATGGGCGAAAGCCTGATCCAGCCATGCCGCGTGTGT
GAAGAAGGTCTTCGGATTGTAAAGCACTTTAAGTTGGGAGGAAGGGCAGTAAGTTAATACCTTGCTGTTT
TGACGTTACCGACAGAATAAGCACCGGCTAACTCTGTGCCAGCAGCCGCGGTAATACAGAGGGTGCAAGC
GTTAATCGGAATTACTGGGCGTAAAGCGCGCGTAGGTGGTTCGTTAAGT

>33_1
ACCTGCCTGTAAGACTGGGATAACTCCGGGAAACCGGGGCTAATACCGGATGGTTGTCTGAACCGCATGG
TTCAGACATAAAAGGTGGCTTCGGCTACCACTTACAGATGGACCCGCGGCGCATTAGCTAGTTGGTGAGG
TAACGGCTCACCAAGGCGACGATGCGTAGCCGACCTGAGAGGGTGATCGGCCACACTGGGACTGAGACAC
GGCCCAGACTCCTACGGGAGGCAGCAGTAGGGAATCTTCCGCAATGGACGAAAGTCTGACGGAGCAACGC
CGCGTGAGTGATGAAGGTTTTCGGATCGTAAAGCTCTGTTGTTAGGGAAGAACAAGTGCCGTTCAAATAG
GGCGGCACCTTGACGGTACCTAACCAGAAAGCCACGGCTAACTACGTGCCAGCAGCCGCGGTAATACGTA
GGTGGCAAGCGTTGTCCGGAATTATTGGGCGTAAAGGGCTCGCAGGCGGTTTCTTAAGT

>33_2
ATCTGCCTGGTAGTGGGGGACAACGTTTCGAAAGGAACGCTAATACCGCATAATATTTCGTCCTACGGGA
TTCGAAAGCAGGGGACCTTCGGGCCTTGCGCTATCAGATGAGCCTAGGTCGGATTAGCTAGTTGGTGGGG
TAATGGCTCACCAAGGCGACGATCCGTAACTGGTCTGAGAGGATGATCAGTCACACTGGAACTGAGACAC
GGTCCAGACTCCTACGGGAGGCAGCAGTGGGGAATATTGGACAATGGGCGAAAGCCTGATCCAGCCATGC
CGCGTGTGTGAAGAAGGTCTTCGGATTGTAAAGCACTTTAAGTTGGGAGGAACGGGCAGTAAGCTAATAC
CTTGCTGTTTTGACGTTACCGACAGAATAAGCACCGGCTAACTCTGTGCCAGCAGCCGCGGTAATACAGA
GGGTGCAAGCGTTAATCGGAATTACTGGGCGTAAAGCGCGCGTAGGTGGTTTGTTAAGG

>35_1
ACCTGCCTGTAAGACTGGGATAACTCCGGGAAACCGGAGCTAATACCGGATAGTTCCTTGAACCGCATGG
TTCAAGGATGAAAGACGGTTTCGGCTGTCACTTACAGATGGACCCGCGGCGCATTAGCTAGTTGGTGGGG
TAATGGCTCACCAAGGCGACGATGCGTAGCCGACCTGAGAGGGTGATCGGCCACACTGGGACTGAGACAC
GGCCCAGACTCCTACGGGAGGCAGCAGTAGGGAATCTTCCGCAATGGACGAAAGTCTGACGGAGCAACGC
CGCGTGAGTGATGAAGGTTTTCGGATCGTAAAGCTCTGTTGTTAGGGAAGAACAAGTGCGAGAGTAACTA
GCTCGCACCTTGACGGTACCTAACCAGAAAGCCACGGCTAACTACGTGCCAGCAGCCGCGGTAATACGTA
GGTGGCAAGCGTTGTCCGGAATTATTGGGCGTAAAGGGCTCGCAGGCGGTTTCTTAAGT

>35_2
ATCTGCCTGGTAGTGGGGGACAACGTTTCGAAAGGAACGCTAATACCGCATAATATTTCGTCCTACGGGA
TTCGAAAGCAGGGGACCTTCGGGCCTTGCGCTATCAGATGAGCCTAGGTCGGATTAGCTAGTTGGTGGGG
TAATGGCTCACCAAGGCGACGATCCGTAACTGGTCTGAGAGGATGATCAGTCACACTGGAACTGAGACAC
GGTCCAGACTCCTACGGGAGGCAGCAGTGGGGAATATTGGACAATGGGCGAAAGCCTGATCCAGCCATGC
CGCGTGTGTGAAGAAGGTCTTCGGATTGTAAAGCACTTTAAGTTGGGAGGAACGGGCATTAACCTAATAC
GTTAGTGTTTTGACGTTACCGACAGAATAAGCACCGGCTAACTCTGTGCCAGCAGCCGCGGTAATACAGA
GGGTGCAAGCGTTAATCGGAATTACTGGGCGTAAAGCGCGCGTAGGTGGTTTGTTAAGT

>36_2
ATCTGCCTGGTAGTGGGGGACAACGTTTCGAAAGGAACGCTAATACCGCATACGTCCTACGGGAGAAAGC
AGGGGACCTTCGGGCCTTGCGCTATCAGATGAGCCTAGGTCGGATTAGCTAGTTGGTGGGGTAATGGCTC
ACCAAGGCGACGATCCGTAACTGGTCTGAGAGGATGATCAGTCACACTGGAACTGAGACACGGTCCAGAC
TCCTACGGGAGGCAGCAGTGGGGAATATTGGACAATGGGCGAAAGCCTGATCCAGCCATGCCGCGTGTGT
GAAGAAGGTCTTCGGATTGTAAAGCACTTTAAGTTGGGAGGAAGGGCAGTAAGCTAATACCTTGCTGTTT
TGACGTTACCGACAGAATAAGCACCGGCTAACTCTGTGCCAGCAGCCGCGGTAATACAGAGGGTGCAAGC
GTTAATCGGAATTACTGGGCGTAAAGCGCGCGTAGGTGGTTTGTTAAGT

>37_1
ACCTGCCCATAAGACTGGGATAACTCCGGGAAACCGGGGCTAATACCGGATAACATTTTGAACTGCATGG
TTCGAAATTGAAAGGCGGCTTCGGCTGTCACTTATGGATGGACCCGCGTCGCATTAGCTAGTTGGTGAGG

TAACGGCTCACCAAGGCAACGATGCGTAGCCGACCTGAGAGGGTGATCGGCCACACTGGGACTGAGACAC
GGCCCAGACTCCTACGGGAGGCAGCAGTAGGGAATCTTCCGCAATGGACGAAAGTCTGACGGAGCAACGC
CGCGTGAGTGATGAAGGCTTTCGGGTCGTAAAACTCTGTTGTTAGGGAAGAACAAGTGCTAGTTGAATAA
GCTGGCACCTTGACGGTACCTAACCAGAAAGCCACGGCTAACTACGTGCCAGCAGCCGCGGTAATACGTA
GGTGGCAAGCGTTATCCGGAATTATTGGGCGTAAAGCGCGCGCAGGTGGTTTCTTAAGT

>37_2
ATCTGCCTGGTAGTGGGGGACAACGTTTCGAAAGGAACGCTAATACCGCATAATATTTCGTCCTACGGGA
TTCGAAAGCAGGGGACCTTCGGGCCTTGCGCTATCAGATGAGCCTAGGTCGGATTAGCTAGTAGGTGAGG
TAATGGCTCACCTAGGCGACGATCCGTAACTGGTCTGAGAGGATGATCAGTCACACTGGAACTGAGACAC
GGTCCAGACTCCTACGGGAGGCAGCAGTGGGGAATATTGGACAATGGGCGAAAGCCTGATCCAGCCATGC
CGCGTGTGTGAAGAAGGTCTTCGGATTGTAAAGCTCTTTAAGTTGGGAGGAACGGGTTGTACGTTATTAC
CGTGCAATTTTGACGTTACCGACAGAATAAGCCCCGGCTAACTCTTTGCCAGCATCCGCGGTAATACAGA
GGTTGCAAGCGTTTTCCGGTTTTACTGGGCGTAAAGCGCGCGTAGGTGGTTTGTTTATT

>38_1
AGCTGCCCGATAGAGGGGGATACCAGTTGGAAACGACTGTTAATACCGCATAATGTTTTGAACTACGGAA
TTCCAAAGTGTGGGACCTTCGGGCCACATGCTATCGGATGCGCCTACGTGGGATTAGCTAGTTGGTGAGG
TAATGGCTCACCAAGGCGACGATCTCTAGCTGGTTTGAGAGGATGATCAGCCACACTGGAACTGAGACAC
GGTCCAGACTCCTACGGGAGGCAGCAGTGGGGAATATTGGACAATGGGCGCAAGCCTGATCCAGCCATGC
CGCGTGTGTGAAGAAGGCCTTCGGGTTGTAAAGCACTTTCAGCGAGGAGGAAGGGGTGCTGTGTTAATAG
TACACACCTTTGACGTTACTCGCAGAAGAAGCACCGGCTAACTCTGTGCCAGCAGCCGCGGTAATACAGA
GGGTGCAAGCGTTAATCGGAATTACTGGGCGTAAAGCGCACGTAGGCGGTTTTTTAAGT

>38_2
ATCTGCCTGGTAGTGGGGGACAACGTTTCGAAAGGAACGCTAATACCGCATACGTCCTACGGGAGAAAGC
AGGGGACCTTCGGGCCTTGCGCTATCAGATGAGCCTAGGTCGGATTAGCTAGTTGGTGAGGTAATGGCTC
ACCAAGGCGACGATCCGTAACTGGTCTGAGAGGATGATCAGTCACACTGGAACTGAGACACGGTCCAGAC
TCCTACGGGAGGCAGCAGTGGGGAATATTGGACAATGGGCGAAAGCCTGATCCAGCCATGCCGCGTGTGT
GAAGAAGGTCTTCGGATTGTAAAGCACTTTAAGTTGGGAGGAAGGGTTGTAGATTAATACTCTGCAATTT
TGACGTTACCGACAGAATAAGCACCGGCTAACTCTGTGCCAGCAGCCGCGGTAATACAGAGGGTGCAAGC
GTTAATCGGAATTACTGGGCGTAAAGCGCGCGTAGGTGGTTCGTTAAGT

>39_1
ACCTGCCCATAAGACTGGGATAACTCCGGGAAACCGGGGCTAATACCGGATAATATTTTGAACTGCATGG
TTCGAAATTGAAAGGCGGCTTCGGCTGTCACTTATGGATGGACCCGCGTCGCATTAGCTAGTTGGTGAGG
TAACGGCTCACCAAGGCAACGATGCGTAGCCGACCTGAGAGGGTGATCGGCCACACTGGGACTGAGACAC
GGCCCAGACTCCTACGGGAGGCAGCAGTAGGGAATCTTCCGCAATGGACGAAAGTCTGACGGAGCAACGC
CGCGTGAGTGATGAAGGCTTTCGGGTCGTAAAACTCTGTTGTTAGGGAAGAACAAGTGCTAGTTGAATAA
GCTGGCACCTTGACGGTACCTAACCAGAAAGCCACGGCTAACTACGTGCCAGCAGCCGCGGTAATACGTA
GGTGGCAAGCGTTATCCGGAATTATTGGGCGTAAAGCGCGCGCAGGTGGTTTCTTAAGT

>40_1
ACCTGCCCATAAGACTGGGATAACTCCGGGAAACCGGGGCTAATACCGGATAATATTTTGAACTGCATGG
TTCGAAATTGAAAGGCGGCTTCGGCTGTCACTTATGGATGGACCCGCGTCGCATTAGCTAGTTGGTGAGG
TAACGGCTCACCAAGGCAACGATGCGTAGCCGACCTGAGAGGGTGATCGGCCACACTGGGACTGAGACAC
GGCCCAGACTCCTACGGGAGGCAGCAGTAGGGAATCTTCCGCAATGGACGAAAGTCTGACGGAGCAACGC
CGCGTGAGTGATGAAGGCTTTCGGGTCGTAAAACTCTGTTGTTAGGGAAGAACAAGTGCTAGTTGAATAA
GCTGGCACCTTGACGGTACCTAACCAGAAAGCCACGGCTAACTACGTGCCAGCAGCCGCGGTAATACGTA
GGTGGCAAGCGTTATCCGGAATTATTGGGCGTAAAGCGCGCGCAGGTGGTTTCTTAAGT

>40_2

ATCTGCCTGGTAGTGGGGGACAACGTTTCGAAAGGAACGCTAATACCGCATACGTCCTACGGGAGAAAGC
AGGGGACCTTCGGGCCTTGCGCTATCAGATGAGCCTAGGTCGGATTAGCTAGTTGGTGGGGTAATGGCTC
ACCAAGGCGACGATCCGTAACTGGTCTGAGAGGATGATCAGTCACACTGGAACTGAGACACGGTCCAGAC
TCCTACGGGAGGCAGCAGTGGGGAATATTGGACAATGGGCGAAAGCCTGATCCAGCCATGCCGCGTGTGT
GAAGAAGGTCTTCGGATTGTAAAGCACTTTAAGTTGGGAGGAAGGGCATTAACCTAATACGTTAGTGTTT
TGACGTTACCGACAGAATAAGCACCGGCTAACTCTGTGCCAGCAGCCGCGGTAATACAGAGGGTGCAAGC
GTTAATCGGAATTACTGGGCGTAAAGCGCGCGTAGGTGGTTTGTTAAGT

>41_1
ATCTGCCTGGTAGTGGGGGACAACGTTTCGAAAGGAACGCTAATACCGCATAATATTTCGTCCTACGGGA
TTCGAAAGCAGGGGACCTTCGGGCCTTGCGCTATCAGATGAGCCTAGGTCGGATTAGCTAGTAGGTGAGG
TAATGGCTCACCTAGGCGACGATCCGTAACTGGTCTGAGAGGATGATCAGTCACACTGGAACTGAGACAC
GGTCCAGACTCCTACGGGAGGCAGCAGTGGGGAATATTGGACAATGGGCGAAAGCCTGATCCAGCCATGC
CGCGTGTGTGAAGAAGGTCTTCGGATTGTAAAGCACTTTAAGTTGGGAGGAACGGGCAGTAAGTTAATAC
CTTGCTGTTTTGACGTTACCGACAGAATAAGCACCGGCTAACTCTGTGCCAGCAGCCGCGGTAATACAGA
GGGTGCAAGCGTTATTCGGAATTACTGGGCGTAAAGCGCGCGTAGGTGGTTCGTTAAGT

>42_1
ACCTGCCCATAAGACTGGGATAACTCCGGGAAACCGGGGCTAATACCGGATAATATTTTGAACTGCATGG
TTCGAAATTGAAAGGCGGCTTCGGCTGTCACTTATGGATGGACCCGCGTCGCATTAGCTAGTTGGTGAGG
TAACGGCTCACCAAGGCAACGATGCGTAGCCGACCTGAGAGGGTGATCGGCCACACTGGGACTGAGACAC
GGCCCAGACTCCTACGGGAGGCAGCAGTAGGGAATCTTCCGCAATGGACGAAAGTCTGACGGAGCAACGC
CGCGTGAGTGATGAAGGCTTTCGGGTCGTAAAACTCTGTTGTTAGGGAAGAACAAGTGCTAGTTGAATAA
GCTGGCACCTTGACGGTACCTAACCAGAAAGCCACGGCTAACTACGTGCCAGCAGCCGCGGTAATACGTA
GGTGGCAAGCGTTATCCGGAATTATTGGGCGTAAAGCGCGCGCAGGTGGTTTCTTAAGT

>43_1
ATCTGCCTGGTAGTGGGGGACAACGTTTCGAAAGGAACGCTAATACCGCATACGTCCTACGGGAGAAAGC
AGGGGACCTTCGGGCCTTGCGCTATCAGATGAGCCTAGGTCGGATTAGCTAGTTGGTGAGGTAATGGCTC
ACCAAGGCGACGATCCGTAACTGGTCTGAGAGGATGATCAGTCACACTGGAACTGAGACACGGTCCAGAC
TCCTACGGGAGGCAGCAGTGGGGAATATTGGACAATGGGCGAAAGCCTGATCCAGCCATGCCGCGTGTGT
GAAGAAGGTCTTCGGATTGTAAAGCACTTTAAGTTGGGAGGAAGGGCAGTAAGATAATACCTTGCTGTTT
TGACGTTACCGACAGAATAAGCACCGGCTAACTCTGTGCCAGCAGCCGCGGTAATACAGAGGGTGCAAGC
GTTAATCGGAATTACTGGGCGTAAAGCGCGCGTAGGTGGTTTGTTAAGT

>43_2
ACCTGCCCATAAGACTGGGATAACTCCGGGAAACCGGGGCTAATACCGGATAATATTTTGAACTGCATGG
TTCGAAATTGAAAGGCGGCTTCGGCTGTCACTTATGGATGGACCCGCGTCGCATTAGCTAGTTGGTGAGG
TAACGGCTCACCAAGGCAACGATGCGTAGCCGACCTGAGAGGGTGATCGGCCACACTGGGACTGAGACAC
GGCCCAGACTCCTACGGGAGGCAGCAGTAGGGAATCTTCCGCAATGGACGAAAGTCTGACGGAGCAACGC
CGCGTGAGTGATGAAGGCTTTCGGGTCGTAAAACTCTGTTGTTAGGGAAGAACAAGTGCTAGTTGAATAA
GCTGGCACCTTGACGGTACCTAACCAGAAAGCCACGGCTAACTACGTGCCAGCAGCCGCGGTAATACGTA
GGTGGCAAGCGTTATCCGGAATTATTGGGCGTTAAGCGCGCGCAGGTGGTTTCTTAAGT

>44_1
ACCTGCCCATAAGACTGGGATAACTCCTGGAAACCAGGGCTAATACCGGATAATATTTTGAACTGCATGG
TTCGAAATTGAAAGGCGGCTTCGGCTGTCACTTTTGGATGGACCCGCGTCGCATTAGCTAGTTGGTGAGG
TAACGGCTCACCAAGGAGACGATGCGTAACCGACCTGAGAGGGTGATCAGTCACACTGGGACTGAGACAC
TGGCCAGACTCCTACGGGAGGCAGCAGTAGGGAATCTTCCACAATGGACGAAAGCCTGACGGAGCAACGC
CGCGTGAGTGATGAAGGCTTTCGGGTCGTAAAACTCTGTTGTTAGGGAAGAACAAGTGCTAGTTGAATAA
GCTGGCACCTTGACGGTACCTAACCAGAAAGCCACGGCTAACTACGTGCCACCAGCCGCGGTAATACATA
GGTGGCAAGCGTTATCCGGAATTATTGGGCGTAAAGCGCGCGCAGGTGGTTTCTTAAGT

>44_2
ATCTGCCTGGTAGTGGGGGACAACGTTTCGAAAGGAACGCTAATACCGCATACGTCCTACGGGAGAAAGC
AGGGGACCTTCGGGCCTTGCGCTATCAGATGAGCCTAGGTCGGATTAGCTAGTAGGTGAGGTAATGGCTC
ACCTAGGCGACGATCCGTAACTGGTCTGAGAGGATGATCAGTCACACTGGAACTGAGACACGGTCCAGAC
TCCTACGGGAGGCAGCAGTGGGGAATATTGGACAATGGGCGAAAGCCTGATCCAGCCATGCCGCGTGTGT
GAAGAAGGTCTTCGGATTGTAAAGCACTTTAAGTTGGGAGGAAGGGCAGTAAGTTAATACCTTGCTGTTT
TGACGTTACCGACAGAATAAGCACCGGCTAACTCTGTGCCAGCAGCCGCGGTAATACAGAGGGTGCAAGC
GTTAATCGGAATTACTGGGCGTAAAGCGCGCGTAGGTGGTTCGTTAAGT

>45_1
ATCTGCCTGGTAGTGGGGGACAACGTTTCGAAAGGAACGCTAATACCGCATACGTCCTACGGGAGAAAGC
AGGGGACCTTCGGGCCTTGCGCTATCAGATGAGCCTAGGTCGGATTAGCTAGTTGGTGGGGTAATGGCTC
ACCAAGGCGACGATCCGTAACTGGTCTGAGAGGATGATCAGTCACACTGGAACTGAGACACGGTCCAGAC
TCCTACGGGAGGCAGCAGTGGGGAATATTGGACAATGGGCGAAAGCCTGATCCAGCCATGCCGCGTGTGT
GAAGAAGGTCTTCGGATTGTAAAGCACTTTAAGTTGGGAGGAAGGGCATTAACCTAATACGTTAGTGTTT
TGACGTTACCGACAGAATAAGCACCGGCTAACTCTGTGCCAGCAGCCGCGGTAATACAGAGGGTGCAAGC
GTTAATCGGAATTACTGGGCGTAAAGCGCGCGTAGGTGGTTTGTTAAGT

>46_1
ATCTGCCTGGTAGTGGGGGACAACGTTTCGAAAGGAACGCTAATACCGCATACGTCCTACGGGAGAAAGC
AGGGGACCTTCGGGCCTTGCGCTATCAGATGAGCCTAGGTCGGATTAGCTAGTAGGTGAGGTAATGGCTC
ACCTAGGCGACGATCCGTAACTGGTCTGAGAGGATGATCAGTCACACTGGAACTGAGACACGGTCCAGAC
TCCTACGGGAGGCAGCAGTGGGGAATATTGGACAATGGGCGAAAGCCTGATCCAGCCATGCCGCGTGTGT
GAAGAAGGTCTTCGGATTGTAAAGCACTTTAAGTTGGGAGGAAGGGCAGTAAGTTAATACCTTGCTGTTT
TGACGTTACCGACAGAATAAGCACCGGCTAACTCTGTGCCAGCAGCCGCGGTAATACAGAGGGTGCAAGC
GTTAATCGGAATTACTGGGCGTAAAGCGCGCGTAGGTGGTTCGTTAAGT

>46_2
ATCTGCCTGGTAGTGGGGGACAACGTTTCGAAAGGAACGCTAATACCGCATACGTCCTACGGGAGAAAGC
AGGGGACCTTCGGGCCTTGCGCTATCAGATGAGCCTAGGTCGGATTAGCTAGTAGGTGAGGTAATGGCTC
ACCTAGGCGACGATCCGTAACTGGTCTGAGAGGATGATCAGTCACACTGGAACTGAGACACGGTCCAGAC
TCCTACGGGAGGCAGCAGTGGGGAATATTGGACAATGGGCGAAAGCCTGATCCAGCCATGCCGCGTGTGT
GAAGAAGGTCTTCGGATTGTAAAGCACTTTAAGTTGGGAGGAAGGGCAGTAAGTTAATACCTTGCTGTTT
TGACGTTACCGACAGAATAAGCACCGGCTAACTCTGTGCCAGCAGCCGCGGTAATACAGAGGGTGCAAGC
GTTAATCGGAATTACTGGGCGTAAAGCGCGCGTAGGTGGTTCGTTAAGT

>47_1
ATCTGCCTGGTAGTGGGGGACAACGTTTCGAAAGGAACGCTAATACCGCATACGTCCTACGGGAGAAAGC
AGGGGACCTTCGGGCCTTGCGCTATCAGATGAGCCTAGGTCGGATTAGCTAGTTGGTGGGGTAATGGCTC
ACCAAGGCGACGATCCGTAACTGGTCTGAGAGGATGATCAGTCACACTGGAACTGAGACACGGTCCAGAC
TCCTACGGGAGGCAGCAGTGGGGAATATTGGACAATGGGCGAAAGCCTGATCCAGCCATGCCGCGTGTGT
GAAGAAGGTCTTCGGATTGTAAAGCACTTTAAGTTGGGAGGAAGGGTTGTAGATTAATACTCTGCAATTT
TGACGTTACCGACAGAATAAGCACCGGCTAACTCTGTGCCAGCAGCCGCGGTAATACAGAGGGTGCAAGC
GTTAATCGGAATTACTGGGCGTAAAGCGCGCGTAGGTGGTTTGTTAAGT

>47_2
ATCTGCCTGGTAGTGGGGGACAACGTTTCGAAAGGAACGCTAATACCGCATACGTCCTACGGGAGAAAGC
AGGGGACCTTCGGGCCTTGCGCTATCAGATGAGCCTAGGTCGGATTAGCTAGTTGGTGAGGTAATGGCTC
ACCAAGGCGACGATCCGTAACTGGTCTGAGAGGATGATCAGTCACACTGGAACTGAGACACGGTCCAGAC
TCCTACGGGAGGCAGCAGTGGGGAATATTGGACAATGGGCGAAAGCCTGATCCAGCCATGCCGCGTGTGT
GAAGAAGGTCTTCGGATTGTAAAGCACTTTAAGTTGGGAGGAAGGGCATTAACCTAATACGTTAGTGTTT

TGACGTTACCGACAGAATAAGCACCGGCTAACTCTGTGCCAGCAGCCGCGGTAATACAGAGGGTGCAAGC
GTTAATCGGAATTACTGGGCGTAAAGCGCGCGTAGGTGGTTTGTTAAGT

>48_1
ACCTGCCCATAAGACTGGGATAACTCCGGGAAACCGGGGCTAATACCGGATAATATTTTGAACTGCATGG
TTCGAAATTGAAAGGCGGCTTCGGCTGTCACTTATGGATGGACCCGCGTCGCATTAGCTAGTTGGTGAGG
TAACGGCTCACCAAGGCAACGATGCGTAGCCGACCTGAGAGGGTGATCGGCCACACTGGGACTGAGACAC
GGCCCAGACTCCTACGGGAGGCAGCAGTAGGGAATCTTCCGCAATGGACGAAAGTCTGACGGAGCAACGC
CGCGTGAGTGATGAAGGCTTTCGGGTCGTAAAACTCTGTTGTTAGGGAAGAACAAGTGCTAGGTGAATAA
GCTGGAACCTTGACGGTACCTAACCAGAAAGCCACGGCTAACTACGTGCCACCAGCCGCGGTAATTCATA
GGTGGCAAGCGTTATCCGGAATTATTGGGCGTAAAGCTTTTTTTGGTGGTTTCTTAAGT

>48_2
ATCTGCCTGGTAGTGGGGGACAACGTTTCGAAAGGAACGCTAATACCGCATAATATTTCGTCCTACGGGA
TTCGAAAGCAGGGGACCTTCGGGCCTTGCGCTATCAGATGAGCCTAGGTCGGATTAGCTAGTTGGTGAGG
TAATGGCTCACCAAGGCGACGATCCGTAACTGGTCTGAGAGGATGATCAGTCACACTGGAACTGAGACAC
GGTCCAGACTCCTACGGGAGGCAGCAGTGGGGAATATTGGACAATGGGCGAAAGCCTGATCCAGCCATGC
CGCGTGTGTGAAGAAGGTCTTCGGATTGTAAAGCACTTTAAGTTGGGAGGAACGGGTTGTAAATTAATAC
GTTGCTGTTTTGACGTTACCGACAGAATAAGCACCGGCTAACTCTGTGCCAGCAGCCGCGGTAATACATA
GGGTGCAAGCGTTAATCGGAATTACTGGGCGTAAAGCGCGCGTATGTGGTTTGTTAATT

>49_1
ACCTGCCCATAAGACTGGGATAACTCCGGGAAACCGGGGCTAATACCGGATAATATTTTGAACTGCATGG
TTCGAAATTGAAAGGCGGCTTCGGCTGTCACTTATGGATGGACCCGCGTCGCATTAGCTAGTTGGTGAGG
TAACGGCTCACCAAGGCAACGATGCGTAGCCGACCTGAGAGGGTGATCGGCCACACTGGGACTGAGACAC
GGCCCAGACTCCTACGGGAGGCAGCAGTAGGGAATCTTCCGCAATGGACGAAAGTCTGACGGAGCAACGC
CGCGTGAGTGATGAAGGCTTTCGGGTCGTAAAACTCTGTTGTTAGGGAAGAACAAGTGCTAGTTGAATAA
GCTGGCACCTTGACGGTACCTAACCAGAAAGCCACGGCTAACTACGTGCCAGCAGCCGCGGTAATACGTA
GGTGGCAAGCGTTATCCGGAATTATTGGGCGTAAAGCGCGCGCAGGTGGTTTCTTAAGT

>49_2
ATCTGCCTGGTAGTGGGGGACAACGTTTCGAAAGGAACGCTAATACCGCATACGTCCTACGGGAGAAAGC
AGGGGACCTTCGGGCCTTGCGCTATCAGATGAGCCTAGGTCGGATTAGCTAGTAGGTGAGGTAATGGCTC
ACCTAGGCGACGATCCGTAACTGGTCTGAGAGGATGATCAGTCACACTGGAACTGAGACACGGTCCAGAC
TCCTACGGGAGGCAGCAGTGGGGAATATTGGACAATGGGCGAAAGCCTGATCCAGCCATGCCGCGTGTGT
GAAGAAGGTCTTCGGATTGTAAAGCACTTTAAGTTGGGAGGAAGGGCAGTAAGTTAATACCTTGCTGTTT
TGACGTTACCGACAGAATAAGCACCGGCTAACTCTGTGCCAGCAGCCGCGGTAATACAGAGGGTGCAAGC
GTTAATCGGAATTACTGGGCGTAAAGCGCGCGTAGGTGGTTCGTTAAGT

>50_1
ACCTGCCCATAAGACTGGGATAACTCCGGGAAACCGGGGCTAATACCGGATAATATTTTGAACTGCATGG
TTCGAAATTGAAAGGCGGCTTCGGCTGTCACTTATGGATGGACCCGCGTCGCATTAGCTAGTTGGTGAGG
TAACGGCTCACCAAGGCAACGATGCGTAGCCGACCTGAGAGGGTGATCGGCCACACTGGGACTGAGACAC
GGCCCAGACTCCTACGGGAGGCAGCAGTAGGGAATCTTCCGCAATGGACGAAAGTCTGACGGAGCAACGC
CGCGTGAGTGATGAAGGCTTTCGGGTCGTAAAACTCTGTTGTTAGGGAAGAACAAGTGCTAGTTGAATAA
GCTGGCACCTTGACGGTACCTAACCAGAAAGCCACGGCTAACTACGTGCCAGCAGCCGCGGTAATACGTA
GGTGGCAAGCGTTATCCGGAATTATTGGGCGTAAAGCGCGCGCAGGTGGTTTCTTAAGT

>50_2
ATCTGCCTGGTAGTGGGGGACAACGTTTCGAAAGGAACGCTAATACCGCATACGTCCTACGGGAGAAAGC
AGGGGACCTTCGGGCCTTGCGCTATCAGATGAGCCTAGGTCGGATTAGCTAGTTGGTGGGGTAATGGCTC
ACCAAGGCGACGATCCGTAACTGGTCTGAGAGGATGATCAGTCACACTGGAACTGAGACACGGTCCAGAC

TCCTACGGGAGGCAGCAGTGGGGAATATTGGACAATGGGCGAAAGCCTGATCCAGCCATGCCGCGTGTGT
GAAGAAGGTCTTCGGATTGTAAAGCACTTTAAGTTGGGAGGAAGGGCAGTAAGTTAATACCTTGCTGTTT
TGACGTTACCGACAGAATAAGCACCGGCTAACTCTGTGCCAGCAGCCGCGGTAATACAGAGGGTGCAAGC
GTTAATCGGAATTACTGGGCGTAAAGCGCGCGTAGGTGGTTTGTTAAGT

>51_1
ACCTGCCCATAAGACTGGGATAACTCCGGGAAACCGGGGCTAATACCGGATAATATTTTGAACTGCATGG
TTCGAAATTGAAAGGCGGCTTCGGCTGTCACTTATGGATGGACCCGCGTCGCATTAGCTAGTTGGTGAGG
TAACGGCTCACCAAGGCAACGATGCGTAGCCGACCTGAGAGGGTGATCGGCCACACTGGGACTGAGACAC
GGCCCAGACTCCTACGGGAGGCAGCAGTAGGGAATCTTCCGCAATGGACGAAAGTCTGACGGAGCAACGC
CGCGTGAGTGATGAAGGCTTTCGGGTCGTAAAACTCTGTTGTTAGGGAAGAACAAGTGCTAGTTGAATAA
GCTGGCACCTTGACGGTACCTAACCAGAAAGCCACGGCTAACTACGTGCCAGCAGCCGCGGTAATACGTA
GGTGGCAAGCGTTATCCGGAATTATTGGGCGTAAAGCGCGCGCAGGTGGTTTCTTAAGT

>51_2
ATCTGCCTGGTAGTGGGGGACAACGTTTCGAAAGGAACGCTAATACCGCATACGTCCTACGGGAGAAAGC
AGGGGACCTTCGGGCCTTGCGCTATCAGATGAGCCTAGGTCGGATTAGCTAGTTGGTGGGGTAATGGCTC
ACCAAGGCGACGATCCGTAACTGGTCTGAGAGGATGATCAGTCACACTGGAACTGAGACACGGTCCAGAC
TCCTACGGGAGGCAGCAGTGGGGAATATTGGACAATGGGCGAAAGCCTGATCCAGCCATGCCGCGTGTGT
GAAGAAGGTCTTCGGATTGTAAAGCACTTTAAGTTGGGAGGAAGGGCAGTAAGCGAATACCTTGCTGTTT
TGACGTTACCGACAGAATAAGCACCGGCTAACTCTGTGCCAGCAGCCGCGGTAATACAGAGGGTGCAAGC
GTTAATCGGAATTACTGGGCGTAAAGCGCGCGTAGGTGGTTTGTTAAGT

>52_1
ACCTGCCTGTAAGACTGGGATAACTTCGGGAAACCGAAGCTAATACCGGATAGGATCTTCTCCTTCATGG
GAGATGATTGAAAGATGGTTTCGGCTATCACTTACAGATGGGCCCGCGGTGCATTAGCTAGTTGGTGAGG
TAACGGCTCACCAAGGCAACGATGCATAGCCGACCTGAGAGGGTGATCGGCCACACTGGGACTGAGACAC
GGCCCAGACTCCTACGGGAGGCAGCAGTAGGGAATCTTCCGCAATGGACGAAAGTCTGACGGAGCAACGC
CGCGTGAGTGATGAAGGCTTTCGGGTCGTAAAACTCTGTTGTTAGGGAAGAACAAGTACAAGAGTAACTG
CTTGCTACCTTGACGGTACCTAACCAGAAAGCCACGGCTAACTACGTGCCAGCAGCCGCGGTAATACGTA
GGTGGCAAGCGTTATCCGGAATTATTGGGCGTAAAGCGCGCGCAGGCGGTTCCTTAAGT

>52_2
ACCTGCCTGTAAGACTGGGATAACTTCGGGAAACCGAAGCTAATACCGGATAGGATCTTCTCCTTCATGG
GAGATGATTGAAAGATGGTTTCGGCTATCACTTACAGATGGGCCCGCGGTGCATTAGCTAGTTGGTGAGG
TAACGGCTCACCAAGGCAACGATGCATAGCCGACCTGAGAGGGTGATCGGCCACACTGGGACTGAGACAC
GGCCCAGACTCCTACGGGAGGCAGCAGTAGGGAATCTTCCGCAATGGACGAAAGTCTGACGGAGCAACGC
CGCGTGAGTGATGAAGGCTTTCGGGTCGTAAAACTCTGTTGTTAGGGAAGAACAAGTACAAGAGTAACTG
CTTGCTACCTTGACGGTACCTAACCAGAAAGCCACGGCTAACTACGTGCCAGCAGCCGCGGTAATACGTA
GGTGGCAAGCGTTATCCGGAATTATTGGGCGTTTTGTGCGCGCAGGCGGTTTCTTAAGT

>55_1
ATCTGCCTGGTAGTGGGGGACAACGTTTCGAAAGGAACGCTAATACCGCATACGTCCTACGGGAGAAAGC
AGGGGACCTTCGGGCCTTGCGCTATCAGATGAGCCTAGGTCGGATTAGCTAGTTGGTGGGGTAATGGCTC
ACCAAGGCGACGATCCGTAACTGGTCTGAGAGGATGATCAGTCACACTGGAACTGAGACACGGTCCAGAC
TCCTACGGGAGGCAGCAGTGGGGAATATTGGACAATGGGCGAAAGCCTGATCCAGCCATGCCGCGTGTGT
GAAGAAGGTCTTCGGATTGTAAAGCACTTTAAGTTGGGAGGAAGGGCAGTAAGCGAATACCTTGCTGTTT
TGACGTTACCGACAGAATAAGCACCGGCTAACTCTGTGCCAGCAGCCGCGGTAATACAGAGGGTGCAAGC
GTTAATCGGAATTACTGGGCGTAAAGCGCGCGTAGGTGGTTTGTTAAGT

>55_2

ATCTGCCTGGTAGTGGGGGACAACGTTTCGAAAGGAACGCTAATACCGCATACGTCCTACGGGAGAAAGC
AGGGGACCTTCGGGCCTTGCGCTATCAGATGAGCCTAGGTCGGATTAGCTAGTTGGTGAGGTAATGGCTC
ACCAAGGCGACGATCCGTAACTGGTCTGAGAGGATGATCAGTCACACTGGAACTGAGACACGGTCCAGAC
TCCTACGGGAGGCAGCAGTGGGGAATATTGGACAATGGGCGAAAGCCTGATCCAGCCATGCCGCGTGTGT
GAAGAAGGTCTTCGGATTGTAAAGCACTTTAAGTTGGGAGGAAGGGCAGTAAATTAATACTTTGCTGTTT
TGACGTTACCGACAGAATAAGCACCGGCTAACTCTGTGCCAGCAGCCGCGGTAATACAGAGGGTGCAAGC
GTTAATCGGAATTACTGGGCGTAAAGCGCGCGTAGGTGGTTCGTTAAGT

>56_1
ACCTGCCTTTATCAGGGGAATAGCCTTTCGAAAGGAAGATTAATGCCCCATAATATATTAAATGGCATCA
TTTGATATTGAAAACTCTTCGGGCCCGGTGGATAAAGATGGGCACGCGCAGGATTAGATAGTTGGTAGGG
TAACGGCCTACCAAGTCAACGATCCTTAGGGGGCCTGAGAGGGTGATCCCCCACACTGGTACTGAGACAC
GGACCAGACTCCTACGGGAGGCAGCAGTGAGGAATATTGGACAATGGGTGAGAGCCTGATCCAGCCATCC
CGCGTGAAGGACGACGGCCCTTGGGTTGTAAACTTCTTTTGTACAGGGATAAACCTTTCCACGTGAATAA
GCTGGGAAGCTGAAGGTACTGTACGAATAAGCACCGGCTAACTCCGTGCCAGCAGCCGCGGTAATACGGA
GGGTGCAAGCGTTATCCGGATTTATTGGGTTTAAAGGGTCCGTAGGCGGACTCGTAAGT

>56_2
ATCTGCCTGGTAGTGGGGGACAACGTTTCGAAAGGAACGCTAATACCGCATACGTCCTACGGGAGAAAGC
AGGGGACCTTCGGGCCTTGCGCTATCAGATGAGCCTAGGTCGGATTAGCTAGTTGGTGAGGTAATGGCTC
ACCAAGGCGACGATCCGTAACTGGTCTGAGAGGATGATCAGTCACACTGGAACTGAGACACGGTCCAGAC
TCCTACGGGAGGCAGCAGTGGGGAATATTGGACAATGGGCGAAAGCCTGATCCAGCCATGCCGCGTGTGT
GAAGAAGGTCTTCGGATTGTAAAGCACTTTAAGTTGGGAGGAAGGGCAGTAAATTAATACTTTGCTGTTT
TGACGTTACCGACAGAATAAGCACCGGCTAACTCTGTGCCAGCAGCCGCGGTAATACAGAGGGTGCAAGC
GTTAATCGGAATTACTGGGCGTAAAGCGCGCGTAGGTGGTTCGTTAAGT

>P putida
ATCTGCCTGGTAGTGGGGGACAACGTTTCGAAAGGAACGCTAATACCGCATACGTCCTACGGGAGAAAGC
AGGGGACCTTCGGGCCTTGCGCTATCAGATGAGCCTAGGTCGGATTAGCTAGTTGGTGGGGTAATGGCTC
ACCAAGGCGACGATCCGTAACTGGTCTGAGAGGATGATCAGTCACACTGGAACTGAGACACGGTCCAGAC
TCCTACGGGAGGCAGCAGTGGGGAATATTGGACAATGGGCGAAAGCCTGATCCAGCCATGCCGCGTGTGT
GAAGAAGGTCTTCGGATTGTAAAGCACTTTAAGTTGGGAGGAAGGGCAGTAAGTTAATACCTTGCTGTTT
AGACGTTACCGACAGAATAAGCACCGGCTAACTCTGTGCCAGCAGCCGCGGTAATACAGAGGGTGCAAGC
GTTAATCGGAATTACTGGGCGTAAAGCGCGCGTAGGTGGTTTGTTAAGT

>E coli
AACTGCCTGATGGAGGGGGATAACTACTGGAAACGGTAGCTAATACCGCATAACGTCGCAAGACCAAAGA
GGGGGACCTTCGGGCCTCTTGCCATCGGATGTGCCCAGATGGGATTAGCTAGTAGGTGGGGTAACGGCTC
ACCTAGGCGACGATCCCTAGCTGGTCTGAGAGGATGACCAGCCACACTGGAACTGAGACACGGTCCAGAC
TCCTACGGGAGGCAGCAGTGGGGAATATTGCACAATGGGCGCAAGCCTGATGCAGCCATGCCGCGTGTAT
GAAGAAGGCCTTCGGGTTGTAAAGTACTTTCAGCGGGGAGGAAGGGAGTAAAGTTAATACCTTTGCTCAT
TGACGTTACCCGCAGAAGAAGCACCGGCTAACTCCGTGCCAGCAGCCGCGGTAATACGGAGGGTGCAAGC
GTTAATCGGAATTACTGGGCGTAAAGCGCACGCAGGCGGTTTGTTAAGT

>B subtilis
ACCTGCCCATAAGACTGGGATAACTCCGGGAAACCGGGGCTAATACCGGATAACATTTTGAACCGCATGG
TTCGAAATTGAAAGGCGGCTTCGGCTGTCACTTATGGATGGACCCGCGTCGCATTAGCTAGTTGGTGAGG
TAACGGCTCACCAAGGCAACGATGCGTAGCCGACCTGAGAGGGTGATCGGCCACACTGGGACTGAGACAC
GGCCCAGACTCCTACGGGAGGCAGCAGTAGGGAATCTTCCGCAATGGACGAAAGTCTGACGGAGCAACGC
CGCGTGAGTGATGAAGGCTTTCGGGTCGTAAAACTCTGTTGTTAGGGAAGAACAAGTGCTAGTTGAATAA
GCTGGCACCTTGACGGTACCTAACCAGAAAGCCACGGCTAACTACGTGCCAGCAGCCGCGGTAATACGTA
GGTGGCAAGCGTTATCCGGAATTATTGGGCGTAAAGCGCGCGCAGGTGGTTTCTTAAGT

>SWI36 biosynthetic gene cluster

```
ATGCAGCAAACCAACCAATTGCCTTTGCAAGGCGTCAATGTCATCGATTTCGGGCAGTACATCGCAGGCCCTGCGGT
CGCGATGATCCTCGCTGATCTTGGGGCCACAGTCGTCCATATCGACCCCCCGGATGGGCCCCTATGGGACAGCCCGG
CAAATGCCACGCTCAACCGCAACAAGCTGTGCATGCGCCTGGATCTGAAGGCGCCAGAGGGGCTGCAGCAAGCTCGC
GAACTCATCTCCCGTGCCGATATCGTGATCGAAAATTTCCGGCCGGGGGTCATGTCCAGATTGGGCCTCGATTTCGC
AGAGCTGCGACGTACTCGCCCTGAGTTGATCACCTTGTCGATTCCAGGGTTTGCCAGCGATGACGAGTTGCGCGCGC
AATGGCGTGCTACCGAGTCGGTGATCGCTTCGGCCTCTGGTGTATTCACCGATATGGGCCAAAACCGCGTGCTGATG
GGCATCAACCCTAGTTTCTCGCCACTTCCCCTGGCGTCAGCGTACGGCACCATGCTGGCTGTGTCGTCTGTGGTACT
TGCTTTGCAGGCGCGTGAAAAGAACGGTGTTGGCGATCAGATCGAAGTGCCCTTGGCATCTGCGGTGATGGAAGGCC
TGTCGTACAACTCGATCAAGATCGATGGTTACCCGCTGCGCTACAAGACCCTGCGTGAGAAGGAGAGCGAGCGTCGT
CGGGCAGAGAACCTGCCGATGAACCTGAGCTACCAGGATTTGCAGGAGTACCTCGACCCGTTCTACCGGACCTATGA
ATGTGCCGATGGCCGTAAATTCTACGCCGTTTGCCCATCGCATCGGGAGCATGCCAAGCGCTGTTTGCAGGCTATGG
GCATCTACGAAGAAATGGTTGCCGCCGGCTTGCCGAAGGTTGATGACCCCTACCTGCCGATTGATCAATGGGAAGGG
AATGCATCCCTGGGCGTTTATCCTTTGCCTGCCGATTGGGCTGCACGTATCTCGGCTCGGATGAAGGACGTGTTCTT
GACTAAAACCGCGGCCGAGTGGGAAGTCATCTTCGGTGAAGGGCGTTTCCCCGGCGCGCCGCACAGGTCTACCCAGG
AGTGGCTCCATGACGAGCACTCGAATACTGCTGGTTTGATTGTCGAGGTCAATGATCGCGAGTTCGGCGTCATGAAG
CAGCCTGGCCCAATTGCCTGGTTGGAAGGTTGTTCAGAACCCATGCTGAAGCCTGTTTCGCGTCGCGATGTGCGCTT
CGAGGAAGCGATGGCAACGCTGTCTGAACGCCCTGTACGTGCATTGCCGCCTGCCAAGCTCGACAAGCCTGCCGGTT
GGCTGGAAGGTGTCAAGATCCTCGACCTGACGAACGTGATCGCAGGGCCGCACTCGACATCGTTCCTGGGCCGCTTC
GGGGCAGACGTCATCAAGCTCGACCCTGTCGTGCCCAATTATGATCCGTGGAATACCGTGGTCTTCGGCATGTCGTC
GGCACGTGGCAAGGAAAGTGTTCTGGTTGATCTGAACAAGCCCGAAGGGCGGGACGTTTTCAATCGTCTGGTGCGGG
AGGTCGACGTGATCGTCATGAACGCGCCTGATCGCCAACTGGCACCGCTGGGCCTGGATGAGGCGAGCCTGCAGGCG
GTGAACCCTGGTGTGATCTTCTGTCAGCTTGACTGTTTCGGCGGCCCACGTCGCGGCCCTCGTACAGATTATCTGGG
GTATGACGACCTGATCCAGGCCACCACCGGCATCATGCTGCGCTTTGGTGGCGGCATGGAAACGCCGGAAGAACATG
CTCACGTTGGCACGATCGATGTGATGTGCGGGTTTGCTGCTGCGCTGGGCGTGGCAACAGCGCTTTATCGCAAGCAT
CACTCCGGGCAAGCTTACCGTGCTCGCACTTCACTGGCATCACTGGGCAATCTGGTACAGATCCCGTTCTGCTACGA
CTATTCAGGCCGGGCGCCCTTCAATGAGCCGTCCGGGCGGGACGTGGTTGGCTACAGCGACCTTTCCCGGTTCTACA
AGACGGCGGATGGCTGGATCTACCTGGATGCCTGCGAACAGGAGTTGCACAAGTTCGCGGCAATCGATGAGCTCAGC
GGGCTGGCGGCGGCCACTGATCGTGTTGCCTACCTGAAATCCGTCATCGGCCGCATGAGCAGTTCGGGTCTTCAAGC
GCGTTTGCAGGCTGCCAACATAGCTGCTGCGGTACCGGACAACATCGATCACCTGCGCAGCCAGTACAGCCGTACCG
CTGATGGCCTGCCGGGTACTGAGAATGGCAGCTACTCCTTCAGCATCTACAGCGACCATCCGAGCGGTCATCGGGTT
ACGCAGCTGGATCCTTATGCCATTCGCCCGCGGGTAGCGCGAATTCGAGCGCTGCCACCCGCTGAAAAGTTTGGCGC
TTCGACTCGCAAGGTATTGGGCCGCTACGGCTATACCGAGGCGCAGATTCAGTCGCTCCTGGACGCCGGCGCAATCG
GTGATTCCTGGAGTGAGGAATATCTGCCCAGCTGACTCCTGAAGGCTGGTCAAGGGGTGCGCCTTCGAGAGGTCCCC
CTGGCTTGTCGACTTACCGTGCAAGCGAACACGAGCCAAAGGCTGCTCGCGGGCGCGTTCATGCCCAAGAAATGAGA
TAGAGCCATGACTTACAACAGCATCCTCCCACGTATCCTGCAAACCGGTGCCGGTGCCAGCCAGCAAATCCACCAGG
TCCTGGCCAGCCTCGGCTGCCATAAGCCACTCATCATCACTGACCGAATGATGGTTGAGTTAGGTTATGCCGGTCGT
ATCAGCGCCGTTCTGGCAGAGCACGCAATAGACGTCGACGTGTTCTCCGACACGGTCCCCGAGCCTACTGTCGCTTC
GATTCAAGCCGGCGTAGCAACCGCCAGGGAGGGAGGCTATGACAGCATCATCGCACTGGGCGGCGGAAGCCCGATCG
ACAGCGCCAAAGCGATTGGCATCCTGGCCAAGTTCGGCGGGGTCATGCGCGATTACAAGTTCCCGCGCAATGTCACC
GAAATGGGTTTGCCGATCATTGCCATTCCAACGACTGCCGGCACTGGCTCCGAAGTCACCCGTTTCACCATCATCAC
CGATGAGTCGACTGACGAAAAAATGCTGTGCGTCGGCGCCGGCTTCATGCCTGTCGCAGCACTGGTCGACTATGAGC
TCACGCTCTCGTTGCCGCCTCGCGTGACGGCGGACACCGGTATCGATGCGCTGACGCATGCCATCGAGGCTTACGTC
AGCAAAAAAGCGAATCTGTTCAGCGATGCCCAGGCACTGGCCGCCATGCGCCTGATCGGGCCAAACCTTCGCAAGGT
CTACCACGACGGGGCCGACCGTGTTGCGCGAGAGGCGGTCATGCTGGGGTCGACCCTTGCAGGTCTTGCTTTCTCGT
CTGCGTCGGTTGCTTTGGTGCATGGCATGAGTCGTCCCATCGGTGCCGCATTCCATGTGCCTCATGGCTTGTCGAAC
GCCATGCTGCTCCCCGAGGTGACGGCCTTTTCAATCCCCGCTGCACGTGACCGGTACGCGGATTGCGCGCGTGCAAT
GGGCGTCGCTACTGAAAGCGATACGGATCAATCCGCAGTTGAAAAACTGCTGGAAGAGTTGGTGGCTATCAACGACG
AGCTGAATGTTCCCACCCCGGCACAATTCGGAATTGATCGCGAAAAGTTCTTTGAACTGATGCCAACCATGGCCGCT
CAGGCGCTGGCTTCCGGTTCACCTGGCAATAACCCGCGTGTGCCTACCGAGGCTGAGATGGTCGAGCTTTACCGCAG
```

CCTTTGGTAGTCGAACAGCAACGCAATACTTGAATAACAAAAAGTGGAGTTTCACATGAATACTGTCGGTCATCTGA
TCAACGGCGAAGCCGTTCTCGAATCCAAGCGCACCCAGGATGTTTTCAACCCCGCCACCGGCCAAGTGGCTCGCCAG
GTTGCGTTGGCATCAAGCCACACGGTAGAGCACGCCATCGCTACTGCCGAAGCCGCTTTTCCAGCCTGGCGCGACAC
GCCCCCTATCAAGCGGGCCCGCATCATGTTCCGCTTCAAAGAGCTCCTGGAACAGAACGCTGATGAGATCTGCAAGC
TGATTGGTGAAGAGCACGGAAAAATTTCTCATGATGCGGCGGGCGAACTGCAGCGAGGGATTGAGAACGTCGAGTAC
GCCTGCGGGATCGCCGAACTTCTGAAAGGCGAACACAGCAAGAATGTCGGCCCGAACATCGATGCCTGGAGCGAGTT
CCAGCCGCTGGGCGTGGTAGCAGGCATCACGCCGTTCAACTTCCCCGTGATGGTACCCCTGTGGATGATTCCAGTGG
CTATTGCGGCGGGGAACTGCTTCATTCTCAAGCCTTCTGAGCGCGACCCAAGCTCTACGTTGTTCATTGCCCAATTG
CTGCAGGAAGCAGGTTTGCCCAAGGGTGTGCTGAATGTGGTGAACGGCGACAAGGAAGCGGTCGACATCCTGCTGCG
CGACGCGCGCATTCAGGCAGTCAGCTTCGTGGGGTCGACGCCGGTTGCCGAGTATATCTACGCCACCGCCACGGCCC
ACGGCAAGCGTTGCCAGGCACTGGGTGGGCCAAGAACCACGCGATCATCATGCCAGATGCCGATATCGACAATGCG
GTTAACCAGCTCCTGGGCGCTGCATTTGGCTCGTCCGGTGAGCGCTGCATGGCGCTGTCAGTCGCCGTTGCCGTGGG
TGACGCGGCAGCTGATGCGTTGATCGCCAAAATGAAAGAAGCCATGAAAAACCTGAAGGTGGGCGCTTACACCGACA
GCAAGAATGATTTCGGCCCGGTGATCACCCGGCAGCACCAGCAAAAAGTGGTCGGCTACATCAACAGTGCTGAAGAG
CAGGGTGCCACGGTGGTTGTCGATGGACGTGAGCCAAGGGTCGCAGGCTACGAGGAAGGCTTCTTCGTCGGTGGCAC
GTTAATCGATCACGTTACCCCAGACATGCAAAGCTACAAGGAGGAGATCTTCGGCCCGGTTCTGCAGGTCATGCGTG
TCAACAGCATGAAGGAAGCCATGGCGCTGATCGACACACACGAGTACGGCAATGGAACGTGCATCTTCACCCGAGAC
GGCGAAGCAGCTCGCTACTTCTCCGACAACATCAAGGTGGGCATGGTCGGCATCAACGTTCCTCTGCCTGTGCCCGT
CGCTTACCACAGCTTCGGCGGTTGGAAGCGCTCGTTGTTCGGTGACTTGCATGCCTACGGGCCGGATGGCGTCCGGT
TCTACACCCGACGCAAGACCGTCACCCAGCGCTGGCCGTCGTCAGGTGTTCGGGAGGGCGTTGAGTTCTCCATGCCG
ACGATGAAGTAAAGGCGGGACAAGATGTGTGCCTGACTGCCGTCGGGCACACATCTTTGTGTGGTGTTAAGGCTCAC
GCGTAGTCGTGCTGCCAGGGTTGTCATGTGCGGTTTTAGGTTTGTTTGGCTGTGCCGCTGAAATAATAATTAAATAA
AATTAGAGGCGAATATGAATAATAAGCACAATATATGTTCGGGAAAGGCTGGCGGCCTTTCATTTCTCCAAGGGACT
CGATCCGTTCGGGCAGGGGTTGCAATTCGTGCTTGACGTATTGGTATTCAACAGATGCTTGTTGTTGAGTCGAACGT
TCTGAGGAGATATGCCAAATGAGTAAGTTAAAAGAGGGTGCCTCATTGGATGGTCGTGTATTTTGGCCATCGATTGC
AGTGATACTCGGCGTCACGATTCCACTTATCGCATTTCCTGAGGCTGGGAATGCGGTGATAAATCAGATGTTTGCGT
TTGCGACCGGGAAGTTTGGCTGGCTCTACCTGGTTTCCGGTTTGATGACTGTCGTCTTCCTGTTGTGGATGGCGTTC
GGGCGCTACGGAAATATACGGCTTGGGCGAAAGAGTGATGCGCCAGAGTTTTCCTACTTTAGCTGGGTCGCGATGAT
TTTTTGTGGCGGTATCGGCATAGCCATTGTCAACTGGGCCTGGGTTGAGCCGATTTATTACTTTAATGGGCCTCCTT
TGAATGTGGCTGCACACAGTCGTGAAGCGGCAGAGTGGGCGTTGACGTATGGGCAGTTCCACTGGGGGTTGACCCCG
TGGGCATTTTACTGCTTGCCGGCATTGCCCATCGCCTATTCGATGTATGTACGCCGACAGCCTGGCGTAAGATTGTC
GGTTGCTGCACGTGGCGTCTTGGGCGAGAAGTCGAATGGGTGGTTGGGAATCACACTTGATACGGTGGTGGTTTTCG
GGATTGTTGGCGGTGTGGGAACATCCCTAGGGTTGGCCGTCCCGCTGGTCTCAACCCTGGCCAGCGACATTTTTGGA
ATACAGCAGTCCTTCGCATTTGACATGTGCGTTTTGGCGCTCTGGACATTGATGTTCGGCATGAGTGTCTGGTTTGG
GCTGAGCAAAGGGATAAAGATACTTAGCGATATAAACGTCTATTTGGCTTTTCTGTTGCTTTTGTTCGCTGCCGTTG
TCGGCCCAACCTTGTTGCTCATCAACGGTTGGGCAAACAGCCTGGGCCTGATGCTCAACAACTTCATCCACATGAGC
CTGTGGACTGACCCTGTTGCGAAAGGTACGTTCCCTCAGGATTGGACCATTTTTTATTGGGCCTGGTGGATTGCTTA
TGCGCCCATGATGGGGTTGTTTGTTGCGCGTATTTCTCGAGGGCGCACAATCAAAGAGCTTATTTTGGCTGAGCTTG
TGTGGGGAAGCCTGGGGTGCTGGGTTTTCTTTGCGGTCTGGGGAGGCTACTCGCTGAATCTTCAGCTCACAGGCGAG
CTGGACGTCAGTGCGATTTTGTCACAGCAGGGTATACCGGCGGCTGTACTCGCCATTTTAAAAACAATGCCATTTTC
AAGTCTTGTTATTGGCGCTTTCGTTCTGCTTTGCTTTGTGTTCCTGGCGACAACGCTTGATTCGGCAGCTTATGTGC
TGGCGTCAGTGACATCGCGTGAGCTCAGCGGCTATGAAGAACCTCAACGCTGGCTACGGATTGTATGGGCGCTGTTA
CTGGCGGTGATTGGCATCGGTCTTCTCAAAGTAGGCGGGCTGAAAGCTGTTCAAACGTCTACCATTGTCGTTGCGCT
GCCTTTGATACCTGTGCTTGGCGTATTGACCTGGTCGCTACTGCGCATGATTCGTGCCGACCTCACGCATGAACTGG
CGTCCAGAGAAATTGTCCTGGAAGACGCGGTGACACTTGCCGATCTGCCGGAGGTTGATGTTTCGCGGGCGGTTGAA
GCTGTTTTGGTTTCGACTCATCCTGAACCTAAATATTAAAGATTGACCTCGTGCTCTATTCGCAAAGGCCTAGGGTT
TCCTAGGTCTTTGGTTTTCTCGCCGTCGCGCGGGTGAAGAGCGATCGAGTGTTCCCACTCAGGGCGATGCAAGCCTG
TGTGGAGGGCAATGTCATGCGGCGGAGCCATAAGCAAAACAAACCCTTTGGGGTGTTTATTCTGATTTTTAGATGGT
GGGGCGACTGAGTATATTGATGGGTTTTTTCTGTGGTGTAGAGCGTTGTTTGTGTCGCAAAATGCTTTGTAATGTCG
CTGGCGATCAACTGGTCTTTTGTGATGTGTCTTATGTTTTCGCAAACCGTTATCTACGTTGTAGGCAACGAGCTTTT
CACTTATTTCGATAAGCGGGCTGCGAGAGAACGCCTGGTTTTAGCAACTCCCAGCCCGAGAACGTTCGTTCAGCCTA
CTGCCCGTATGCTGATGTGGTTGCCAGATTTGCACGGGCCCATGCCTCAGCGGTCGTGACCTGAATCGACTGCTGCG
CATTGAAAGTGCCGGCTTTAGGCCATGCCACGCCTCTGCCTTGGGCGAAGACAGCGCGGTACTTCTTGATGTGATGC

GTTGGGTCTTCCTCCAACTCTTGGAGCAAGTACGGCACAGTCCACACGTTGCGTTTGAACGGGCGGCCCAGTACACG
TTCGAGGAGGCTCGCAACCTCTCCGTACGTCACGGTGTCGCCCGAGAGGTAGACAATCTGATTGCGGAAGCGAGGTT
CGAAGAAAACGATCTCCGCTGTCAACGCGCCAATGTCATCTGGCGTCGTGAGCGTCACGCTGGTCTCAAGACTGCCT
AGCGCGTTCACCGTATCGTTTTCGAAGTCAACCACCTCGAATACTGGCTCGAACAGAAAGCTGGTGAACATGCCCGT
CGAGATGATTACCCATTCAGTTTTATCCTGGGCTCGAAGCAATTCGCGGACATCAAGCTGAGCATCGAACAAATCCT
GAGGGCTGCCCCGACCGATTACCTCGAAGTCGACACCAAACTGCCACGGGAAGTAGCGCTTCACACCAGCCTTCAGG
GCGGCGGTGGCCAGCTTCATTGGGGTTTCCCGGCCTGCAACCATGCCTGCACAGCCTATTACGGTATCGAAACGTGC
GAATACTTCAGCCAGCTGATCGATTGAGTCATTCACGAGATCGGCAGCCACCATCTGGATCCCGAGGCCCCGAAGCT
CATCGATTTTGACCTTTTTCTCGGGCACTTGCGTGTTGATGGTCGAATCTCTGAGCAGGACGCTGATTGTGGAACCG
GGCGCGCGTTTTGCTAGTCGCGCAAGATTGCGCAGCACCGGCAGCCCAAGCTCACCGGCGCCTAGAACGAGGATGGA
TTGGGGGGGAAGCTGGGTCACTTCGGTCATGATTTCTCCTGAGAACTGTTGCCTGTAAGATGAACAAATGCTTGCAC
GATTAGGCAACGTTCGTAAGAAGGCACACCTGTGATACTTGAGAAAGCAATGACTGATCAGGAGGCGCTAAGCCAGG
CAGAGACGATTTGCCGAACGCTTAGAGAAGACGATGATGGCGTCAGGCGAGAAGTGCTCGCTCACGCAGGCAGTCGC
TGGTCGTTAGGTATTCTGCATGCCCTGGGGGTATACGGCACGATGCGCCATGCCGAAATAAAAAGGCAGATGACGGG
GGTGACTCAGCGGATGTTAACCAAGACGCTGCGCTCCATGGAGCGGGATGGCCTGGTGCTTCGGCGGGAGTTCGGAG
AGATTCCGCCACGTGTCGAGTATGAGCTCACGCCACTTGGTATGGGGCTGTTGATCCGCATGTCACCTGTCTGGACT
TGGGTTGTCGAGAACGTTGAGGACTTCCGCAAGGCGCGGCGTATTTTCGACAGTCAGGATGACAAGAAGCCAGCGTG
GCAAATACCTGCATCAACACCATTCCATTCAGACGCGGATGACTGAATGTAGCTCACAGGGGGTGAGAAGCCCTGTG
CCCGTTCATGCATGCCAATTGAATCACAGGTCGAAGACCTTGCGATGAGCATGGGCCATGCAGCCGATTTTGATGAT
GCGCCCTCCCAAGCCGGCCATGTAGCCTGCGAAGTGATCGCAGACCAGGTTGCCGTTCCAGGTGCCCTGGAAGTTGC
GCCCATGTTCGTCAGGACGGCTCGGGCTGAAGTGGATGACGTTGGCGTTGACGTCGTGGCTGATGGCGACGCTCCAA
ACCGTGGCGCCAGGCTGGAGGCATTCCTGCACTTTACCTTTATCTGTGCCAAAGCTTGATACTCAATGAGCCGCCGA
CCTTGCTGAATGGGAGATGCCTTTGATGGAGTTACGCCAGCTTCGTTACTTTCTGAGTGTGCTGGAGTGTGGCAGCC
TCGGCAGAGCCGCGCTCGAAACGGGCGTTGGGGTATCTGCGTTGAGCCAGCAACTCACGAAGCTCGAAAGTGAGTTG
AGTACCCGCCTGCTGACACGCTCTAGCGTTGGCGTCACGCCTACCGCGGCAGGTTTGGCATTTGAATACCATGCGCG
CCTGACGCTGCGCCAGGCCGATTACGCCATGCTGGCTGCACGCAGTGGGCGTATGAGTGGGTACGCCAGCGTCGGTA
TGGCACCCACCACGGCTTCGGTGTTGGGGCTGGCGCTGATCGCCAGGATGCGCGAGCGCTACCCTGATATACGTCTT
CATTTGGTGGAAATGCTGTCGGGCTACCTGGTGAACCAGCTCAATACTCGGCACCTGGACTTGGCGGTGTTGTTCCA
GCTGGAGGCTGGCATGCGGCTGGATGCGCGGCCGCTGCTGGAGGAGCGGCTGTTCGCATTGGTTCCGGTAGCCTTGG
TCAAGGCTGACTGGGGGACGTCGCTGACGTTGCAGCAGATCGCCTCGCTGCCCTTGGTGATGCCCAGCGCCCAGCAT
GGTCTTCGCGCCACGTTACGCGTCATCCTTGAGCGCGTCGGATTGGACGCGAATATCGTCATGGAGGTTGATGGCCT
GTCGCTTCTCATGGATTGCGTCAGCGCAGGTCACGCCGCGACGATTCAACCCGGCGCCACTGTGGCCCGGGCCATGC
AGGCAGGCCTGCGGGTGTTTTCAATCGACGATCCGCAAGCTCAGCGTCGCAACTTGGTGGTGAGCCTGGTCGATGAT
GAACTCTCGCCTGCTGCACTTGCCACGCGAATCGTGCTGCATGAAGTGTCCCGCGAATTGGTGCAGCAGGATCGCTG
GCCAGGGGCGCGTTTGCTGTGAGCGCCTGCGAACGACCGTCCCTTTAGAAAAACTGAAGACCTCCCCCCTGCAATCG
TGTTTCGCCCAAGGTACGCGACCCTCTAGAGTCCGGGGTCCGCGAGGAGCTGCCCAACCCTGGCTTCTCGCTCCCTG
GAGGCGACATGATCGATGTACTTGTCATTGGCGGCGGCAATGCTGCCCTCTGCGCCGCGCTGATGGCGCGCGAGGCC
GGCGCCAGCGTGATGCTGGTGGAAGCCGCGCCCCGCGCCTGGCGAGGCGGCAATTCCCAACACACCCGTAACCTTCG
ATGCATGCACGATGCCCCGCAGGACGTGCTGGTGGAGGCTTACCCCGAAGAAGAGTTCTGGCAGGATCTGCTGAAGG
TTACTGATGGCCAGACCAACGAGGCCCTGGCGCGTCTGGTGATTCGTGCCTCGTCCGACTGCCGCGGCTGGATGCGC
CGCCACGGCGTGCACTTCCAGCCCTCGTTGTCGGGTGCGCTGCACACTGCGCGGACCAACGCGTTCTTCATGGGCGG
GGGCAAGGCCCTGGTCAATGCTTATTACCGCAGCGCCCAGCGGCTTGGTGTGCAGGTGCGCTACGACAGCCCGGTGT
GCGATCTGGAGTTGCGCGATGGACGCTTCATCGCTGCCCACCTGGCGGCCCGCGACGTCGACGGGCGGCGCCTGCCC
GCCGAGCGCATCGAGGCGCGCAGTTGCGTGCTGGCGGCCGGCGGCTTCGAGTCCAATCGCCAATGGCTGCGCGAGGC
CTGGGGGCAGAATAATCGCGGCGAGTGGCCGGCGGACAACTTCCTTGTGCGGGGCACGCGCTTCAACGACGGCGCGC
TGCTGCGGCGCATGATCGACCTAGGTGCCGACACCATTGGCGACCCGACCCAGGCCCACATGGTCGCCATCGATGCC
CGCGCACCGCTGTACGACGGCGGTATCTGCACGCGCATCGACTGCGTGTCGCTTGGCGTGGTGGTGAACCGCGACGG
CCAGCGATTCTACGACGAAGGCGAGGATTTCTGGCCCAAGCGCTATGCCATCTGGGGGCGCCTGGTGGCCCAGCAGC
CTGGCCAGGTGGGGTTCTCGATCATCGACCAGAAGGCCCTGGGCCGCTTCATGCCACCGGTCTTCCCTGGCAGCCGC
GCCGACAGCCTCGAGGCCTTGGCTGTAGCCCTCGGACTGCCGGTGCCTGCGTTTCTCGAGACCCTGCGCGCCTACAA
CCAGGCATGCCGCCCCGGCACGTTCGACCACACCCAGCTGGACGATTGCCGTACCGACGGCGTGCAACCGGTCAAGA
GCCATTGGGCCTTGCCGATCGATAAGCCGCCGTTCTTCGGTTACCCGCTACGTCCGGGGGTGACCTTCACCTACCTC
GGCCTGCGCACCGATGCCACGGCCGCCGTGCATTTCGCTGGGCGGCCCAGTCCCAACCTGTTCGTCGCCGGCGAGAT

GATGGCCGGCAACGTGCTGGGCAAGGGCTACACCGCCGGTGTCGGCATGTCCATCGGTACCGCGTTTGGCCGCATCG
CCGGCACGCAGGCGGCGGCTGCCGCCGGCCATTGTATTTCCCCCCTGGAGGCCCAGCATGACTGTGCAAATGCCTGA
TCCCCACGTGTTCGAACCCGCCCCGCCACAGGCCGAGCTGATCCCGCTGCTGAACCTGGACGAGCAGGAGGTGGACC
GGCAGATGCGCATCTGCAACGCCTGCCGTTATTGCGAGGGGTTCTGTGCCGTGTTCCCGGCGATGACCCGTCGACTC
GACTTCGGCAAGGCCGACATCCACTACCTGGCCAACCTCTGCCACAACTGTGGCGCCTGCTTGCACGCTTGTCAGTA
CGCTGCACCGCATGCGTTCGCGGTGAACGTGCCGCAGGCCATGGCCAAGGTGCGCGGGCAGACCTACGCTGAGTACG
CCTGGCCGGCCCGTTTCGGGCAGCTCTACCGGCGCAATGGCACCTTCGTCGCCGTGGCGTTGGCACTGGCGCTGTCG
CTGTTCTTGCTGCTGGCCTTGCAGGTCAACGGCACTTTGCTGCCTGGGCGGCTGGCCGGAGATTTCTACGCTGTGTT
CCCGCACAACACCTTGGCGCTCTTGTTCGGTGGCGTGTTCAGCGCTGCCGGGGTGGCGCTGGGCGTGGCGTTGAGGC
GCTTCTGGCGCACGGTGTCGCCCACCCAAGCGCGGCAACCGGCCAACGGTGCGCTGCCGGAGGCGGCGAGCGCAGTG
CTGACGCTCAAGTATCTGGATGGCGGCCATGGCCAAGGCTGCAACAACGCCGACGATCGCTACACGCTGTGGCGTCG
ACGCTACCATCACTTGACCTTCTACGGCTTCATGCTGTGCTTTGCCGCCACCGTGGTCGCCACCGGATATCACTACC
TGCTGGGCCTGCATGCGCCATACCCGCTGCTGAGCCTGCCGGTAGTCCTCGGCACCCTCGGCGGTATCGGCCTGATC
GTCGGGCCAGCGGGCCTGCTGGCGTTGAACCTGCGCCGCGCGCCAGAGCATGGCGACGCCGCGCAGCGCCCCATGGA
CCGTGCGTTCATCCTGCTTCTGCTGTTGGTCAGCACGACCGGTATGGCGTTGCTGGCCCTGCGTGACACCGAGGTGA
TGGCGATCCTGCTGGCCGTGCACCTGGGCACCGTCATGGCACTGTTCGTGACCTTGCCCTATGGCAAGTTCGCCCAT
GGCCTGTTCCGCAGTGCCGCGCTGCTGAAGTTTGCGATCGAAAAGCGCCGGCCCGACCCTCACAGCTTGGGCGGGGA
CTGAACCCTGCCGGGGATGCGATGCGACACCGTGTGTTCGGCCTGGCGAGCAACGCAGTGGCCGCCGTGCTGATCTC
GCTGTCCGAGGGTGCCTGAGAACGCGAAACCCTGCTGCGTCGCCTGAAGGGTCCGCCGAACCTCATCGCCACGGCCG
ACCGAACCGTACACCCCGCATTGACACAAACGCCTACCTGGCATCAAGCGATGCCGGGTGGGGAAGGGGGCCGTCTA
CCCGTCGGGCGGCCACATAGACAATAAGAACAAGAGAACAAACCATGCTCGCACTTCTGGGCCTGATCATGGTGGTG
ACCTTCACCTACCTGATCATGAGTAAACGCCTGTCCCCGATCGTCGCGCTGACCATCGTGCCGATTGTTTTCGCCCT
TGTTGGCGGTTTCGCGCCAGACCTGGGCAAGATGATGCTCGATGGGCTGAAAATGGTCGCACCGTCTGCCGCGCTGC
TGCTGTTCGCCATCCTGTTTTTCGGCCTGATGATCGACGCCGGCCTGTTCGACCCGCTGATCCGCAAGATTCTCAAG
CGGGTGAATGGCGACCCGATAAAGATCGCCATCGGCACCGCCTTGCTATCGCTGCTGGTGGCGCTGGACGGTGACGG
CACCACGACCTACATGATTACCTGCGCCGCCATGCTGCCGCTGTACAAGCGCATCGGCATGAACCCGATGATCCTGG
CGACCGTATCGATGCTGTCATTGAGCATCATGAGCGGCATGAGCCCGTGGGGTGGTCCCGCCACCCGTGCCATTGCA
GCGCTCGGCCTGGATGCGACCGAATACTTCATTCCGATGTTGCCCACCGTCATCGGTGGGGCGGCATGGGTCGTGTT
CACCGCCTACCTGCTGGGCCGTGCCGAGCGCCGGCGTATCGGCAATATTGCCCTGGAGTCCGGTGGCGGCAACTGCT
ACATCAAGGAAATCCTCGGCGACAGCCCGCACAAGCGCCCGCGCCTTGCCTATGTGAACCTGGTGCTGGTGATCGCC
GTGATGACCGCGCTGGTGCTGGGGCTGATGCACGCGGCCATCCTGTTCATGATCGGTTTCGTCGCCGCACTGATGAT
CAACTACCCGCAGCTGGACCTGCAGAAAGAGCGCATCCTCGCCCATTCGGGCAACGCCATGACCGTGGTGCTGCTGG
TGTTCGCCGCGGGCATCTTTGCCGGTATTTTCTCGGGTACCAAGATGGTCGATGCATTGGCCCAGACGCTGGTGGAC
TGGATTCCGGAAGCGTGGAGCCACTGGTTCCCGCTGGTGGTGGCGATGACCAGCATGCCGCTGACCTTCGTGCTGTC
CAACGACGCGTACTACTTCGGCGTTGTGCCGATCCTGGCCAATGCCGCGGCCGCGTACGGCATCGACCCGGTGGAAA
TCGCCCGCGCCTCGGTGCTTGGCCAGCCGGTTCACCTGATGAGCCCGCTGGTGGCCTCGACCCTGCTGCTGGTGGGC
ATGGTCGACCGCGATATCGGTGATTTCCAGAAAGCCACCTTCAAGTGGGCCGTGCTCACTTCTCTGGTGATCACTGC
GCTGGCACTGCTGACGGGCGCCTTGTCCTTCATTGTTTGAAGCACTCAGGGCGCCGCTTGCGGGTACGGTGATATCC
CGCTTCTGGAACTATCGTGCTGTTTCAAGCAATCGATTTTGTCATTTTACGACCCCTCTCCAAGCCCCATTGAGCTG
CATAAATCAAAGGTTTCAGGGCGCGTCGGGATTTCTCGCAAAAAGCACTGATGGCACGTTGACTCTCCCCTTAGGGG
GAGACTTTAGACTCCCGGCCCCCGATAGATCTGGCCTTAAAACAGCTTTTTTTGGCCCGATGACTTTATTCATTCAA
TGCGGCTTTTGTAGGGAAACACTCAATGAATAATCCGTTGGAATTGGACAGCGTTATCAGCAGTACACAGGATATTC
TTGCGCAGTTGCTGGTGCTGGATAGGGGCGACGTGACAGAGCACAGCAGTATCGTTGACGACCTCAGCGCCGACTCG
CTGGACATCGTCGACCTGAGCTTCCAGCTGGGCCGTCAGTACGGTTGCACCTTGCCGAAAACCAGCGTGCTGGATCA
CGCGGTAGCGGTGTTCGGCGACGCCACCCGGTTTGTCGAAAATGGCCGAATCACGCAGGACGGCGTGGCCTTGCTGG
AGCAGAGCCTCAGCGCTTATGCGCCGGGCCAGCTTCACGTGGGCATGCAGCCGGGCGATGTCTTCTCGGCCACCACC
GTGCGGAACTGGGCGCAGCAATGCCATAACGTGTTCAACCACCTCCCCGAAACCTGCCCTGAGTGTGGCGCGGCTCA
TGCCCAACTCAATGAACGCCAACAAGTGGTGTGTGGCGGTTGCAGTGCTCGCCTGACCCCTCTGGACGGTGACTCGA
TCTCGCGTCTGTTGGTTGAGCAGTACGCGGCCGCCCAGTTGAAGGCTTCGGCGTAGGGGGTGCCTTGATGCAGGCCC
GAGAGGTTCTGGTCACCGGCTATGGACTGGTGGCTCCCACGGCACTGGATGCGGCCTCGCTGTTCACGACGATCAGC
GAAAACCGTTCCTGCATTGGCCAGCACCCGGCGTTTGTCGAACTGGGGTTTGCGAACCCGGCGGCCGGATTTATCGA
TGATCAACAATGGCAGGTCATCGCGGCGGCCTTTCCGGGCGACCTGCAGACCACCTCCCGCCAGGAATGGCTGGCGC
ATTACGTGGCCCGTCAGGCGCTGGCCCATGCCGGGTTGGCCGACGGCGCATTTGCCCGAGTCGCCAGCGGTATTTTC

GTCGGGGCCAACAAATATTGCGTGAGCAGTGATTTGCGGGACGCCAGCCGTTACATGGACGATCAGGGGCGGGTCGA
TCTCGACCAGTTCCTGGATAACCACACCCTCAGCGGTGGGGCGTTTGCCCGACGGGTTGATCAGCAAACCCGCCATC
TCGCCGAATGGCTGGGTGTCCGTGACCATATCTCCACGCATTCCGATGCCTGTGCCGCCGGCACCATGGCCATCGGC
AGTGCCTATCGAGCGATCGCCCGTGGCGAGATCGACCTGGCGTTGTGTGGCGCGGTGGAGTTGATGGCCCATGAATT
GTCCTACTACAGCTTTGATGGGCTGGGCGCACTCTGCCAGCGCGTGGACTTTTCCCCACAAGAACAGAGCCGGCCGT
TCATGCCGGACCGCTGCGGTTTTGTGCTGAGTGAAGGGGCGGCGCTGTTGGTGCTGGAATCCCGCGAGCATGCCGAG
CGTCGTCAGGCCTGTTCGCTGGGCCGGGTGCTGGGGTACGCGAATCTGTGCGAGGCAGAGAAAATCACCTCCAGCAG
CCGCGACGGCAGCAAGTATGCCGCCTGCATGACGGCGGCCCTGGCCGATGCCGAACTGACCAGTGCGGCTGTGGACC
ATGTGAATGTTCACGGTACGTCGACACAGGCCAATGACCGTTGCGAGGCCCTGGGCCTGGTGCAGGTCTTCGGCGAA
ACCCTGGAGCAGATGACGTTCACCGCCAACAAATCTGCCGTAGGGCATTCGTTGGCCGGCAGTGGTGCCATTGAGGC
GGTGCTGTCGTTGATGAGCATTCAGTTGGGCGTGGCCTTGCCGACCCTCAATTTTCAGCCGGAAAAATCCGAGTTTC
CGGCGTTGAAATTCCTTTGCGAACCGTTGCAGCAACCGATCAATGTCGTGCTCTCCAACTCCTTTGGGTTTGGTGGC
GTGAACAGTTCGTTAGTGCTGGGCAGGGCATGACATGACCAGATCCAATTCAACCGCTGTTTATATCACTGCGGCGT
CGGTACTCAACGCCGCCGGTACTGAATGTGCGCCTCGTGACAGTCAACCTCAGGCCGCGCAACCCCTGGATTTCGAC
CCGGCACGTCTGGCCCATTGTGTCGCAGCACCCGCCTTGCCGGCGGGTGTGTACGACCGCAGACTGTCCCGCAGCCT
CGAGCCTCAGGGCGCGCGCCTGCTGTATTGCGCCGGCCGTCTCGCCGAGACCTTGCGTGGGCTGGAACTGCCCGACG
AGCGGATCGCATTGATCGCTGCCATTCCGGAAGTGGATGGCCCCAGTTCGTGCTGGGATGCAGTGCAGGCCATTGGC
GCACAACCGGCAGATCTGTTGGCGCAATTCTTTGCCCACACACCGCCGCTGCATGCGCTGATGATGCTCAACAGCAG
CGTCATGGCCCACGCGGCTGAGGCCTTGCGTTGCAAGGGGCCCATGGGCGGTTTTTGCTCCCAGGGCAATGCGGGGC
TGGATGCTTTGATGGAGGCCGCTGCCCATCTGAGCGAAGGCCGTGCCGATGCTGCCGTGGTCGTCAGCAGCAGTCCG
AACATCACCCCGGCGTTGTACCTGCGTGACGCTACTCACCCACCTGTAAACCCCGGCCCGTGGGTTTTCGGCGAAGG
TGCTGCGGCACTGTTGCTGACGACCAACCCAGGCAGCGGTGCTCAATCTGCGTTGCGTATTGCCGGGTTTGCCCGTG
GTTACAGCGCGCAACCCGAGCGTGGACTGGCGGTTGGTCGCGACGTCATTGAGCGTGCTTTGAGCAACGAAAAACTG
TGCCTCAGCGATGTCGGGCAAGTCGTCGCCGATGCACAGGATCCGCAGATTGCCGCGTTGTTTAGCCTATCGGGGCA
CACCCTCGAACACTCCAAGGCGTTGCTAGGCGACTTGGGCGCCAGCGCCCTGCTCAGCGAAATTGCTGTGACCTGGG
CACTGGATTGCAGCCGTTACACGCTGTTGCTCGAACATAGCCGAGGCGGGCATTGGGGCGCGGTACTGCTGGCCAGA
ACGTCGTCGGACACAGAGACAATGGAGAGTTACCCATGAGCCCCACACGGATCGTGATCACGGGCATGGGCGCCGTC
ACCGGCTTCGGTTTCGAGTGGCAGTCCATGTGGCAAAGAATGTTGGCCGGCGAGCATTGCATTCGGCCATGGCAACC
GGAAGGCGTCGAGGCGGGTGCGTTCCCCGTGCGTTACGCAGCGCCCGTGGATATGGGCTTGATGCCGGCACACTTGA
AAGACCATCCGGCCTGGGGATTGTCGCTGGAAAAACGCACCCGCTTTGGCTGGGTCGCGGCCACCCAAGCCATTGCC
GATAGTGGTCTGAGCCCCGAACAGTTGCGTGGCGCGGCGGTATTGTCAGCGTCCGGCGCACCTGCTCATCGTTTGCA
GGACATGCTGCTCAGCCTGGCGGATAACCCGGCGCAGGCGCCGTCCTGGGCTCAATTGATGGCGCGACAGGCGCAGG
TCGATCCTGACAGTTCACTGTGTCAGAGCAACGACCGCCTTGCGCGGGTGATTGCCGATGGCATCGGCAGTGAAGGC
CCGGTGATCAATGTCAGCAGCGCCTGCGCGGGAGCGTCCCAGGCCATCGGCAACGCATTCCAGATGATCCGTCGCGG
CGAGATCGAGCTGGCCATTGCCGGGGGCGCCGATTCGGTGTTGAGCCTGGACACCATGACCGCGCTGTATCTGCTCG
GCGCCGCCAGCAGCGAACAGCGTTGGGGCACCGAACTGTGTCGGCCGTTCGACCGCCATCGCAGTGGCTTGATTGCG
GGTGAAGGTGGTGGTTTCGTAGTGCTCGAAACCCTCGAACGGGCGCTTGCCCGAGGGGCAACGCCGTACGCCGAAGT
GCTGGGTTTCGGCAGCAGCCTGGATGCCTACAAAATCACCGCACCCGACCCGCAGGGCCGGGGCGCGGTGCTGGCGA
TGCAGGCGGCCCTGACGGATGCCGGGCTGGCGCCGCAACAGGTCGATCTGATCAATGCCCACGGTACCTCGACACCC
CTCAACGATGCCGCCGAAACCCTGGCGATCAAGACCCTGTTCGCCGAGCGCGAACATTATCGGCGGTTGCTGGTGAC
GGCCAACAAATCCCAGTTCGGCCACCTGATTGCGGCCGCCGGAGCACCGGAGTTCATGCTCACGGCCCTGGCCTGCC
GGGATGATCGAGTCACGCCGACCCTGAACCTGCAGGATGCCGACGAACAATGCGACCTCGACTACTGCGCCCACCAA
GCGGTCAAGCGCAAGGTCGACGTTGCCCTGAGCAACTCCTTCGGTTTCGGCGGCCTCAACACTTCGCTGGCGCTGGG
CAAGTACCGGGAGGCGCCGCGATGAAACAGGCGGTCGCGATTGCCGGTGCGGGTTGCGTGTTGCCCAGCGGCTGGGG
TGTCGAGTCTTTCTGGGCGGCCGCAACCGAAGGGCGCAGTGCAATTACGGCGCTGAACGCCCGACGGTTCAGCAGCG
AACGCGTGGTGGCGTTCGGGCAAATTCAGGATCAGGACCACCAACGCAGCCGACAAGACCTTGCGCAAAACCTTCAG
CGTTACTGCACGCCGGCAGTGATCTGGGGTGTCAGCGCGGTACGTCAGGCGTTGGAAGAAGCCGGCCTGGCCGATGA
GCAGCCGGATGTCCGTTTCGGTTTGTATTGCTGTCAGGGCGGCTACACCCATCCGTCTCTGGAATCCTATGCCGAGT
TGTTGCACGGCTGTCGGCAGGACGGCGTCGCCGACATGGCGGCGTTCGCCAAACGGATCCTGCAAGACCGTGCCCAG
GACCCTTTTCTGGTGCTCAAAGGCTTGAGCAACTGCTTGCTGGGTGTCACCAGCCTGGCGCTGAAACTGGTGGGCGA
GTGCAACGCCTATATGCAAGGTGTCGCCGGCAACCTGGCAGCCTTGCGTGAGGCTACGGCCGCTTTGCAGGATGGCC
GAATTGACGCGGCAATCGTTGTCGGCGCGGGCAGCGAGCTGGATGCCCTGGCGCTGTCCGGGTTGGTCCAGGCGGGT
GTCATCAGCGCCAACGGCTCGAACACGCTGCTGCCGTTTGACCTTCGGGGCACTGGCGGCATTGCCGGGGAGGGCGC

GGCAGCGCTGGTGCTGCGCCGGCGCGAAGACCTGCCGGCCGGTCCTCAGGCGTGCCTCGCCGACATGACGGCCCATG
CCTCCCTCGGTCGCTTGAGCCTGCCCGACAGCCCAACCGATGTGTTGGTGTGCAGCGGCACCGGCGATGCGCACAAA
GACCGCGTGTTGTGCCAGACGCTGGCCCTTGCGCGCGCCTCGCACATCACCAGCGGGTTGGCGATCAACGGCATTCT
CAGCGCGGCGCCGAGCCTGGTGGATCTGACTCTTGCGCGTTGCGCATTGCTGGCCCAAAGCGTGCCGCCGATTGCCG
GTTTGCAGCAACCAGTGGCGAACCTGCCGTTCATTCAGGGCGCGCCCCACGCCGCGAGCCTTGCGCATTGCCTGGTG
CTGAACCGCGACGACAACGGATTCAGCGCCGCCTATCAAGTGCTCTACAGCGCAAAGGTCACTCAGGACTGCCGCTG
ACTACCGCCTCTTTTTTACTTTCAAGGGATATGACTGTCATGGATTATCGCGATTTCGTTCGGCCCAAATTCGTCGG
CCTGCTCCAGGCACTGGGCCTGGAGTGTGAATTTGAGCGCGCCCTCGGTAGCCAGTTGTTCTACCGCAACCCCAAGG
GCGATATGGTCACGGTCACCGACTTTCTCGGCGGTTACGGTGCGGCACTGTTCGGCCACAACGACCCACAGTTTGTC
GATCAGCTATGTGCGCTGCTGCGCAGCGACGTGCCGTTCAACGCACAAATGTCGATCCGCGGTGCGGCCGGGCAATT
GGGGCGAGCGCTCAGCGACGCCTTCAATCGAGAACTGAAGAACACCGAACGTTATATTTCGACTTTCTCCAACAGCG
GCGCGGAGGCGGTGGAGATCGCCGTCAAGCACGCGGAGTTCCGTCGCCAGAAGAGCCTGCAAAAGCAGTTCGACGAC
ATCGATTTCACCCTGGCCAGCCTGACCGCCAGCGAACACGCTTACCGCGAACTGGATGTGGCCGACCTCGACCTGCC
TGCAGGTGTCCTGCCGGCCACCCTTGACAGCGTGACCTTGCGTCAGGTGGTTGAAGCCGTTCGCCAACACAACCTGG
CGCAGTTGCACATCGAACCGGTATTCGTCGCCCTGCGCGGCAGCTTCCACGGCAAATTGGTCAATACCGTGCAACTG
ACCTATGGCCGTCAGTACCGCGCGCCGTTTTCCCGTTTTGGTCTGAACGTCGAGTTCATCGACCCACAGCAGCCCCA
TCAGTTGCAGGAATTGCCGGCACGGCACACGCACCATTGGTTGAGCCTGCAATGGGACGGCGAACACCTGCACGTGC
TGCAACTGCCGTTCAGTGCGATTACCGCTGTGTTGATGGAACCGATTCAGGGCGAAGGCGGTATCAACGAGTTCGCG
GCCGAGTTCTATCTGGGGCTGCGCAAGCTGTGTAACGAGCAACAATGCCCGCTGGTGGTCGATGAAGTGCAGTCGGG
TTTTGGTCGCGCCGGGACGTTTCTTGCCAGCAGCCAGTTCAACCTGCAAGGCGACTATTATTGCCTGTCCAAGGCCC
TCGGCGGCGGCTTGATGAAAATCGCCGCGACGGTGATCCGCAGCAGCCACTACGAAGGCGAGTTCAGCTACATCCAC
AGCTCGACCTTCGCCGAGGACGACCCGTCCTGCCATATCGCGCTGTCGGCATTGCGCCGGTTGTTCGCCGATGACGA
CGCGATGCTCAAAGACATATGCACCAAGGGCGCCTACCTCAAAGCCTCGCTCAATGAACTGAGGATGGCCTATCCGG
ATGTGATCGCCGATGTGCGCGGGCGTGGCTTGCTGGTGGGGCTGGAATTGCACGATTTGAGTGTCAGCAGCTCGCTG
GTACAGGCGTCGGCCCAGTACAATGATGCCTTGGGTTACTTGATCGCCGGTTACCTGTTGCAATTCGAAGCGTTGCG
GGTGGCACCGTCGGGCAGCAACTCCAACGTGATTCGCCTGGAGCCGCCAGCCTGTATCACCCTGGGCGAAATCGACA
AGCTGATCGCTGCCCTGCAACGGGTCTGCGACATGCTGCGCCGTGGCGATGCCTTGCCGTTGGCGGCGGGTATCTGC
GCGGACTCGATGCCCGCCGTGCCAGCCCGTCAGGACGATTTTCGGGTGGCCGAGCCCGTTGAAAACAGGGATGCCAA
GGTCGAAGTCGTCGCTCGGGTAGCGTTCATCAACCACCTGATCGATGCCGATATCCTCGGTGACGTCGATCCCTCGC
TGGCAGCCCTGAGCCCGGAACAAAAACGCACGTTCATCAATCGCACCAAGCCTGAACGCCGCGCTGTGCCAGTCGGC
CCGGTGATCATTCGGTCGCGCCTCGGCATGGCGGTGGAGTTCACGTTATACCCGCTGTGCATGGACTCGGATGCCAT
GGCCGAGTACATCCGCAGCGGTGACCTGGACAGCATTCGCGAAGAAGTCGGCAGGCGTGTCACCGATGCCCGCGCCG
ATGGCTGCAGCATCGCCGGCCTCGGGATGTACACCTCGATCGTCACCAACAATTGTCAGGCGCTGAAGATTGCCGAC
ATGGCGCTGACGTCCGGCAACGCCCTGACCATCGGCATGGGGCTCGAAGCCATCGAGCAAGGCTGTGTACAGCAAGG
TTTGGCACTTTGCGAGCAAACCGCTGCCGTTGTCGGTGCCGCCGGTAACGTTGCGTCGACCTATGCCTCGGTGCTGT
CCGCCACCGTCGATCACCTGATTCTGATTGGCAGCGGTCGTGACGGCTCGGTTCGGCGCCTGGAGAAAACTGCGCAA
CAGATTTACGCCGACGCGGCCCGTTCGATCCTCAAAGGCACGGCCGAAAACGACCGCTTGGCCCAGCGACTGCTGAC
CCTTGATGGTTTTCGTGGCTTGTTGCACTCACATGGCCAAAAAGCCGACCTCGGTTTGCACATCGCCCGTCTGGTGG
ATGAGCGCCTTGGCGCGAACGCCTTCATCACGGTCACCAACGATCTCGACGCCATCAAGGGCGCACGGATTGTCCTG
TGCGCTGCCAATGCGCCTCAGCCGTTCCTCGGTGCCGAACATTTTGCCGAGCGCAGCGTGATTTGCGATATCGCCGT
GCCGCTGAACGTCCATCAAGACCTGCCGAGCCAACGCGAAGACGTGCTCTACATGCACGGCGGCATCGTCCAGACGC
CGTTCGACGACGGCCTGGCGCCCAACGTGCGGGCCTATCTGAAAAAAGGTCAGCTGTATGCGTGCATGGCCGAGTCG
GTGTTGATGGGCTTGTCTGGCATGAGCCAGCACGGCTCCTACGGGGATATCAGCCGTGAGCAAGTTCAGCAGGTCCG
CGCACTGGCCACGACCCACGGTTTCACCCTCGCGCAATTCAAAACTCAAAACTCACTTTAAGGAGCGGTCATGACTC
AGAAAATAGCTGTTGTGACCGGCGGCAGCCGTGGCATTGGCAAGTCCATCGTGCTGGCCCTGGCCGGCGCGGGTTAT
CAGGTTGCCTTCAGTTATGTCCGTGACGAGGCGTCGGCCGCTGCCTTGCAGGCGCAGGTCGAAGGGCTTGGCCGGGA
GTGCCTGGCCGTGCAGTGTGATGTCAAGGAAGCGCCGAGCATTCAGGCGTTTTTTGAACGGGTCGAGCAACGTTTCG
AGCGTATCGACTTGTTGGTCAACAACGCCGGTATTACCCGTGACGGATTGCTCGCCACGCAATCGTTGAGCGACATC
ACCGAGGTCATCCAGACCAACCTGGTCGGCACGTTGTTGTGCTGTCAGCAGGTGCTGCCCTGCATGATGCGCCAACG
CAGCGGGTGCATCGTCAACCTCAGTTCGGTGGCCGCGCAAAAGCCCGGCAAGGGCCAGAGCAACTACGCCGCCGCCA
AAGGCGGTGTAGAAGCATTGACACGCGCACTGGCGGTGGAGTTGGCGCCCCGCAACATCCGGGTCAACGCGGTGGCG
CCCGGCATCGTCAGCACCGACATGAGCCAAGCCCTGGTCGGCGCCCATGAGCAGGAAATCCAGTCGCGGCTGTTGAT
CAAACGCTTCGCCCGGCCTGAAGAAATTGCCGACGCGGTGCTGTATCTGGCCGAGCGCGGCCTGTACGTCACGGGCG

AAGTCTTGTCCGTCAACGGCGGATTGAAAATGCCATGAGCCAGTTACGCACGATTGTGATTACCGGCGCGGCCAATG
GTATTGGCCGCGCCGTCGCGGAAAGTTTTGCAGCCCAGGCCGAGCACCTTTTGATCTTGCTTGATCGGGATCTGGAA
ACCTTGCAGAGCTGGGTCACCGAGGGCGAATTTGCCGCTCGCATCGAAACCCATCAAGCGGACATCGCCGATCTCGC
CAGCCTGCAACTTCTGTTCAAGGGCCTGGCCAACCGGGTCGGCTTTGTCGATGTGCTGGTCAACAGCGCCGGGGTCT
GCGACGAGAACGAACCCGAAGACCTCGACAACTGGCACAAGGTCATTTCGATCAACCTCAACGGCACCTTTTACGTC
ACCTCGTTGTGCCTGCCACTGATAGCCGATGGTGGGCGGATCGTCAACATGTCGTCGATCCTCGGCCGAGCGGGCAA
GGTCCGCAACACCGCTTACTGCGCCTCCAAGCACGGCATCATCGGCATGACCAAAGCCTTGGCGCTGGACCTGGCGC
CACGGCGAATCACCGTCAATGCCATCCTGCCCGCCTGGATCGACACACCGATGCTGCAAGGCGAATTGGCGGCTCAA
GCGCGCATCGCCGGGATCACACACGAGCAGATCCTGCGCAATGCCAAGAAGAAGTTGCCCCTGCGGCGCTTCATTCA
GGGCGACGAAGTGGCGGCCATGGTTCGTTATCTGGCGAGCCCGCAGGCTGGTGGTGTCACGGCGCAAAGCCTGATGA
TCGACGGCGGTGCCGGGTTGGGAATGTAG
